# Digital Interventions for Emotion Regulation in Children and Early Adolescents: Systematic Review and Meta-analysis

**DOI:** 10.2196/31456

**Published:** 2022-08-19

**Authors:** Sally Reynard, Joao Dias, Marija Mitic, Beate Schrank, Kate Anne Woodcock

**Affiliations:** 1 Centre for Applied Psychology University of Birmingham Birmingham United Kingdom; 2 Faculty of Science and Technology University of Algarve Faro Portugal; 3 Algarve Centre of Marine Sciences Faro Portugal; 4 Institute of Systems and Computer Engineering: Research and Development Lisbon Portugal; 5 Die Offene Tür Research Group for Mental Health of Children and Adolescents Ludwig Boltzmann Society Karl Landsteiner University of Health Sciences Krems Austria; 6 Department of Psychiatry and Psychotherapy University Hospital Tulln Tulln Austria; 7 Institute of Mental Health University of Birmingham Birmingham United Kingdom

**Keywords:** emotion regulation, digital interventions, youth, systematic review, meta-analysis, children, early adolescents, serious games, training, biofeedback, mobile phone

## Abstract

**Background:**

Difficulties in emotion regulation are common in adolescence and are associated with poor social and mental health outcomes. However, psychological therapies that promote adaptive emotion regulation may be inaccessible and unattractive to youth. Digital interventions may help address this need.

**Objective:**

The aim of this systematic review and meta-analysis was to synthesize evidence on the efficacy, feasibility, and acceptability of emotion regulation digital interventions in children and early adolescents aged 8 to 14 years.

**Methods:**

Systematic searches of Web of Science, MEDLINE, PsycINFO, EMBASE, Education Resources Information Centre, ACM Digital Library, and IEEE Xplore up to July 2020 identified 39 studies, of which 11 (28%) were included in the meta-analyses (n=2476 participants). A bespoke tool was used to assess risk of bias.

**Results:**

The studies evaluated digital games (27/39, 69%), biofeedback (4/39, 10%), virtual or augmented reality (4/39, 10%), and program or multimedia (4/39, 10%) digital interventions in samples classified as diagnosed, at risk, healthy, and universal. The most consistent evidence came from digital games, which reduced negative emotional experience with a small significant effect, largely in youth at risk of anxiety (Hedges *g*=–0.19, 95% CI –0.34 to –0.04). In general, digital interventions tended to improve emotion regulation, but this effect was not significant (Hedges *g*=0.19, 95% CI –0.16 to 0.54).

**Conclusions:**

Most feasibility issues were identified in diagnosed youth, and acceptability was generally high across intervention types and samples. Although there is cause to be optimistic about digital interventions supporting the difficulties that youth experience in emotion regulation, the predominance of early-stage development studies highlights the need for more work in this area.

## Introduction

### Background

Emotion regulation difficulties are prospectively associated with negative social outcomes [1] and psychiatric disorders in youth [2]. This is particularly significant considering that half of all lifetime psychiatric disorders begin by the age of 14 years [3], and a recent large-scale meta-analysis reported 14.5 years as the worldwide peak age of psychiatric disorder onset [4]. The malleability of affective neural circuitry is heightened from late childhood through early adolescence [5,6]. Hence, this is a period of particular interest for harnessing adaptive emotion regulation strategies, which may support positive social outcomes and psychological well-being [7-9]. Proximal social input that manipulates the environment in a positive manner, such as targeted intervention, can improve emotion regulation ability [10,11]. Digital interventions may constitute efficacious, accessible, and attractive interventions in youth [12]. However, there is no systematic understanding of existing emotion regulation digital interventions and their efficacy in youth. Consequently, the aims of this systematic review and meta-analysis were to present a comprehensive understanding of the extant evidence on digital interventions that target emotion regulation in youth to provide recommendations for this emerging field.

### Emotion Regulation in Youth

Emotion regulation is operationalized as the attempt to recognize positive and negative emotional reactions in ourselves and to increase or decrease these in ourselves or others [13,14]. The Extended Process Model of emotion regulation provides a framework of emotion regulation stages (identification, selection, implementation, and monitoring) and strategies in relation to an emotional goal (refer to the study by Gross [15] for a comprehensive account) and is consistent with the way many extant digital interventions for emotion regulation have been designed.

The developmental trajectories of improvements in different stages of the Extended Process Model are not equivalently linear [16]. In line with this, neural networks implicated in emotion regulation follow a pattern of protracted refinement and reorganization through synaptic pruning and myelination through late childhood and adolescence into early adulthood [5,11]. This may explicate adolescents’ heightened sensitivity to rewarding experiences, increased experience of negative emotions, and variability in affect compared with young children and adults [16-18]. Indeed, strengthening emotion regulation relies on improved connectivity between affective and reward-processing networks and prefrontal cognitive control networks [19]. This key developmental process is malleable and influenced by internal and external factors such as hormonal changes and social relationships [10,11]. Critically, this malleability is somewhat heightened from late childhood through adolescence [5,6].

### Emotion Regulation Interventions in Youth

Proximal social input that manipulates the external environment in a positive manner, such as targeted intervention, can improve emotion regulation ability in youth [10]. Traditional face-to-face psychological interventions that aim to promote adaptive emotion regulation in youth include cognitive-, emotion-, and mindfulness-based talking therapies such as cognitive behavior therapy (CBT) [20], rational emotive behavior therapy [21], and dialectical behavior therapy [22]. These are facilitated by a psychologist in 1:1 sessions or small groups, depending upon the needs of the individual and available resources. CBT aims to reduce the selection and implementation of maladaptive cognitive emotion regulation strategies (eg, rumination) and instead promote adaptive ones (eg, cognitive reappraisal). CBT is effective in adolescent populations [23]. However, such therapies are time, money, and personnel intensive [24], and youth may experience traditional programs as unattractive because of perceived mental illness [25,26] and related help-seeking stigma [27]. Negative attitudes toward traditional approaches may be reflected in poor engagement, as evidenced in dropout rates of up to 75% [28].

Preventive emotion regulation programs that are wider reaching than traditional therapies focus on the engagement and education of the caregivers of youth [13]. Such programs encourage explicit tangible learning and practice of adaptive emotion regulation strategies [13] either in the classroom [29-31] or through home-based socialization [32,33]. Although highly encouraging, these interventions may not be accessible or appropriate for all young people. For example, disadvantaged youth demonstrate an increased potential for withdrawal from mainstream services [34] through which wider-reaching interventions are provided.

### Digital Interventions in Youth

Mental health digital interventions for youth have attracted a number of recent systematic reviews and meta-analyses [12,35-37]. The most common types of digital interventions include virtual and augmented reality, internet therapy, biofeedback and neurofeedback, digital games, and web-based programs [12,35,36]. Although this is an emerging field, preliminary evidence suggests that digital technologies may constitute clinically effective, economical, accessible, and attractive interventions for mental health problems in youth [12]. Moreover, the internet is widely accessible, even to populations who may not have access to support using traditional means [38].

However, previous reviews have focused on a broad age range, encompassing childhood, early adolescence, late adolescence, and early adulthood. This may not permit the understanding of the impact that digital tools have within childhood and early adolescence—a critical period of brain development [5,6] and time of newly increased interest in, and engagement with, web platforms [39].

### This Study

Considering emotion regulation–specific digital interventions, an example is the small number of freely available mobile apps accessible through the UK National Health Service (NHS) digital technology library for mental health. These claim to support well-being through heart rate biofeedback, breathing techniques, and gamified calming strategies. Such freely available interventions are born out of national health care provision policy implemented by the NHS, which is driven by clinical need and economic considerations; yet, there is no empirical research to provide evidence for the efficacy of these apps. Furthermore, no extant systematic reviews or meta-analyses present such evidence for emotion regulation digital interventions in children and early adolescents.

In parallel to the question of efficacy, the study by Bevan-Jones et al [40] highlighted concerns regarding levels of user engagement, uptake, and adherence in mental health digital interventions for youth. This is discussed in line with best practices in digital intervention development in which active involvement of key stakeholders (eg, early adolescents) is recommended to facilitate the feasibility of digital interventions as well as their acceptability [40]. A systematic understanding of how far digital interventions for emotion regulation have achieved feasibility and acceptability and how these are evaluated is also important. In this systematic review and meta-analysis, we aimed to evaluate the extant evidence base for the use of digital technologies to improve emotion regulation in children and early adolescents and provide recommendations for the progression of the field.

### Research Questions

We formulated the following research questions:

1. What are the characteristics of digital interventions that have been evaluated in terms of the efficacy and feasibility of their impact on emotion regulation in children and early adolescents?
2. How efficacious and feasible are emotion regulation digital interventions in children and early adolescents?
3. What are the experiences of children, early adolescents, and other stakeholders regarding the acceptability of emotion regulation digital interventions that evaluate efficacy or feasibility?

## Methods

Details of the protocol for this systematic review and meta-analysis were registered on PROSPERO [41].

### Information Sources and Search

Web of Science, MEDLINE, PsycINFO, EMBASE, Education Resources Information Centre, ACM Digital Library, and IEEE Xplore electronic databases were used to identify studies. Groups of search terms pertaining to children and early adolescents, digital interventions, and emotion regulation were identified through scoping searches and combined using *OR* (within groups) and *AND* (across groups) Boolean operators and syntax. Search terms and associated Boolean operators and syntax were adapted for different databases as necessary. Refer to Multimedia Appendix 1 for the full search strings for each database. Gray literature searching using Open Science Framework Preprints and OpenGrey electronic databases, as well as forward and backward tracking, was used to identify further studies. An author voluntarily sent 1 study to the authors. Searches were initially run in August 2018 and repeated as a top-up search in July 2020. The initial search was broader than the top-up search. Before the top-up search in July 2020, the inclusion criteria were reviewed. Because of the need to narrow the focus of the review, studies targeting social cognition only and acceptability or qualitative design only, theses, and studies in which samples were aged <8 years or >14 years were excluded. Therefore, at this stage, the social cognition search terms were removed from the search string. The age of 14 years was determined as the upper age limit because of increasing evidence of the need for early emotion regulation intervention efforts from an empirical as well as public health perspective [4,29,30,33,42].

### Eligibility Criteria

Studies were included if they met the following inclusion criteria: (1) used digital technology as an intervention strategy, (2) aimed to improve emotion regulation and associated neurobiological mechanisms, (3) targeted children and early adolescents (mean age between 8 and 14 years), and (4) reported data on the efficacy or feasibility of the digital intervention with or without acceptability data. Studies reporting only acceptability data without corresponding efficacy or feasibility data were not included. Studies meeting these inclusion criteria and published after 2008 in peer-reviewed journals presented in English, German, Portuguese, Spanish, Italian, Serbian, Croatian, or Hebrew were considered for inclusion. Studies published after 2008 were included because scoping searches conducted in August 2018 revealed that emotion regulation digital interventions were developed after 2008. Quantitative and mixed methods studies that used any relevant outcome measure were considered for inclusion.

Studies were excluded if they met any of the following exclusion criteria: (1) not original research paper, extended conference paper, or preprint (ie, book, book chapter, commentary, conference abstract, or conference poster), (2) development or testing of technical intervention component only (eg, statistical simulation without assessment of a psychological variable), (3) animal population, and (4) population with organic neurological disorder, and if they did not meet the inclusion criteria.

Please refer to the Meta-analyses subsection under the Methods section for specific information on meta-analysis eligibility criteria.

### Study Selection and Data Collection

Using the web-based reference management software Covidence (Veritas Health Innovation Ltd), 2 independent reviewers (SR and JS) conducted record screening [43]. Any conflicts between the reviewers’ screening decisions were resolved through consensus, with involvement of a third experienced researcher if necessary. PRISMA (Preferred Reporting Items for Systematic Reviews and Meta-Analyses) guidelines [44] and a bespoke risk-of-bias assessment tool (refer to the next section) were used when designing and conducting the data extraction protocol. A piloted standardized Microsoft Excel table was used to extract data from the included studies for evidence synthesis and risk-of-bias assessment (refer to Multimedia Appendix 2 [45-83] for the extraction table). The first author (SR) completed this task.

### Assessment of Risk of Bias

A tool for risk-of-bias assessment was created using evidence-based information and guidance. This was sourced from the Cochrane Collaboration’s tool for assessing risk of bias in randomized trials [84], Cochrane Methods risk-of-bias web-based library [85], NHS National Institute for Health and Care Research guidance for feasibility and pilot intervention studies [86], and the Newcastle-Ottawa Scale for assessing the quality of nonrandomized trials [87]. Wide-ranging information and guidance were required because of the breadth of research designs and associated methodological characteristics included. Care was taken to feature the risk-of-bias domains relevant to the included studies and questions in the review. The tool consisted of 6 domains: (1) selection bias, (2) performance bias, (3) detection bias, (4) attrition bias, (5) reporting bias, and (6) other bias. The risk-of-bias assessment was conducted independently by 2 two trained reviewers. Any conflicts were resolved through discussion, with involvement of a third experienced researcher if necessary. Refer to Multimedia Appendix 3 for the risk-of-bias tool domains.

### Clustering and Coding of Included Studies

Guidance on the conduct of narrative synthesis in systematic reviews [88] was followed to generate a thematic understanding of the included digital interventions. The included studies were coded and clustered using defined criteria based on the key category of intervention type. The criteria and definitions that were used to cluster the included studies are detailed in Textbox 1. Studies were further coded based on the population type. These were identified as *diagnosed*: children and early adolescents diagnosed with a physical or mental health disorder; *at risk*: children and early adolescents at risk of a mental health disorder (eg, elevated anxiety); *healthy*: typically developing children and early adolescents with no identified diagnosis; and *universal*: no exclusion criteria applied. The studies’ outcome targets were coded based on what they measured. These were *emotion regulation*: recognition of emotions in oneself and the increase or decrease of these emotions; *emotion experience*: negative (eg, frustration) or positive (eg, joy) emotions or symptoms; and *physiological regulation*: brain or bodily signals associated with emotion regulation and emotion experience (eg, heart rate).

Tables were created to summarize the characteristics of the included studies and the efficacy, feasibility, and acceptability data. Within the tables that present the efficacy, feasibility, and acceptability findings, we provide the raw reporting risk-of-bias information at the measure level for information and transparency.

**Clustering of included studies based on intervention type.**

**Cluster and definition**

- Biofeedback: A digital physiological monitoring aid
- Digital game: An electronic game with functions to achieve specific goals, with or without biofeedback
- Virtual reality and augmented reality: A simulated environment (ie, a digital immersion experience with no physical world input); an enhanced reality (ie, a digital sensory component on a live smartphone view)
- Program and multimedia: A program or multimedia application

### Acceptability, Feasibility, and Efficacy

Acceptability and feasibility data were synthesized within intervention clusters with validity and reliability reporting bias and attrition bias information where appropriate. In studies not included in the meta-analyses, within-intervention group before-and-after emotion regulation, emotion experience, and physiological regulation efficacy data were further synthesized with significance and effect size information where available. Hedges g (the summary measure) was calculated in R [89], using the esc package [90] to indicate whether significant observed effects were small (0.2), medium (0.5), large (0.8), or very large (1). In a very small number of studies, it was not possible to calculate Hedges g or convert to it (eg, where only η^2^ value was provided).

### Meta-analyses

Studies included in the systematic review were considered for inclusion in the meta-analytic component if they were randomized controlled trials (RCTs) [91]. Of the 39 studies included in the systematic review, 3 (8%) were not sufficiently homogeneous to other included studies; therefore, they were not included in the meta-analyses. Of these 3 studies, 2 (67%) used informant report only and reported emotion regulation effects alongside emotional expression as a composite score and 1 (33%) implemented a crossover design. In addition, another study did not provide data to calculate effect sizes and hence was not included. Noninferiority RCTs (which compared the intervention to efficacious group face-to-face CBT and hypothesized nonsignificant differences between groups), which are increasingly prevalent in the intervention literature, were included in the meta-analyses. Including noninferiority trials is a more conservative approach in terms of the resultant effect sizes that would be expected; the majority (6/9, 67%) of the other studies included in the meta-analyses used an active control, and some of these were class-based psychoeducation, which has demonstrated beneficial effects. Thus, noninferiority studies were deemed similar enough to other RCTs to be included [92]. It is acknowledged that this may have resulted in the pooled effect being lower than if noninferiority effect estimates had been excluded. Including nonrandomized studies was considered; however, such studies were not reasonably resistant to biases (they were all judged as high risk of bias, inclusive of confounding bias, and varied greatly in methodological design [91]).

Meta-analyses using a very small number of studies may negatively affect the estimation of between-study variance [93]; therefore, a threshold of 4 studies was established as a suitable minimum. Thus, 2 meta-analyses were conducted, focusing on emotion regulation and emotion experience outcomes, respectively. From each study, 1 effect was selected for each meta-analysis to ensure the independence of effect sizes [94]. All the studies used self-report measures; therefore, 1 self-report effect from each included study was selected. Where studies provided multiple self-report effects for each outcome target type, constructs from self-report scales or subscales that were most similar to each other across the included studies were selected. For example, most emotion experience effects measured anxiety across the included studies; hence, where possible, anxiety-based effects were selected for meta-analysis. Where studies used multiple comparison groups, the active control group data were used. Further standardization was facilitated by computing postintervention standardized mean differences only. This is because follow-up data collection was not incorporated into the designs of all meta-analytic studies, and where it was, the length varied greatly across studies. Studies were also coded based on intervention type (biofeedback, digital game, and program and multimedia), population type (diagnosed, at risk, healthy, and universal), training of additional skills (yes or no), use of additional mode of intervention delivery (yes or no), and measure risk of bias (low or high). In addition, dropout rate was included in the results of the meta-analyses.

Analyses were conducted in R [89] using meta-analyses packages tidyverse [95], meta [96], metafor [97], and dmetar [98]. The studies included in the meta-analysis varied somewhat in methodological design; therefore, a degree of heterogeneity was assumed. In line with this, random effects models were applied [99]. The restricted maximum likelihood estimation of tau-squared (*τ*^2^) between-study variance was used because it corrects for negative bias within continuous data (in which large tau-squared is reported when the number of studies and individual studies’ sample sizes are small), unlike the standard DerSimonian-Laird method [100]. The mean and SD of each study’s selected effect was used to calculate Hedges g and its SEs. Hedges g was computed because the commonly used Cohen *d* [101] may demonstrate a slight bias in small studies in which effects are overestimated [102]. In each meta-analysis, study ID was the unit of analysis, and the effect size (g) for each study was the level of analysis [103].

Heterogeneity was estimated using *I*^2^, tau-squared, and the prediction interval (range into which the effects of future studies are expected to fall) because of the possibility that any one measure on its own is inadequate [98]. Specifically, although *I*^2^ is insensitive to increases or decreases in the number of studies, it relies on each individual study’s sample size to predict the amount of variability in the effect sizes *not caused by sampling error* [98,104]. *I*^2^<25% indicates low heterogeneity, *I*^2^=50% indicates moderate heterogeneity, and *I*^2^>75% indicates high heterogeneity [105]. Tau-squared, the between-study effect size variance estimator, is insensitive both to each study’s sample size and the number of studies in a meta-analysis, but the meaning of tau-squared might be difficult to interpret alone [98].

Outlier analyses were conducted to determine whether extreme effect sizes contributed to between-study heterogeneity, using a CI-based approach [98]. Influence analyses were conducted to determine the robustness of the pooled effect estimates using leave-one-out principles [98]. Influential cases were examined in subplots [106]. These revealed how much the predicted pooled effect changed in SD units after excluding a given study, the distance between the value when the study was included versus excluded (the Cook distance), and the covariance ratio [98]. Extreme values were shown in red. In addition, the plots were examined to detect any extreme cases not defined by the Viechtbauer and Cheung threshold [106]. Baujat plots were created to determine each study’s heterogeneity input [107]. Finally, 2 leave-one-out forest plots that ordered studies by *I*^2^ between-study heterogeneity and effect size (low value to high value) were created to provide further evidence of influential studies [98]. As digital games constituted most (9/11, 82%) of the studies in the meta-analyses, additional meta-analyses were conducted using only digital game effect sizes where appropriate.

### Publication Bias

Several steps were taken to investigate potential publication bias, which occurs because of selective publication of significant findings with large effects [98,108]. Particularly in small studies, where very large effects are needed to reach statistical significance, the results are more likely to be statistically significant if their effect sizes are high. First, contour-enhanced funnel plots were examined visually. Contour-enhanced funnel plots, which present color shading linked to significance levels, allow the distinguishing of publication bias from other sources of asymmetry, for example, variable study quality [109]. The Egger test of the intercept quantified funnel plot asymmetry—a statistically significant result (*P*<.05) determines asymmetry [110]—although this possesses low statistical power in <10 studies [91]. Where the Egger test was significant, the Duval and Tweedie trim-and-fill method was used to estimate the actual effect size had the *missing* small studies been published [111]. Missing studies were imputed into the funnel plot until symmetry was attained.

## Results

### Study Selection

**Figure 1:**
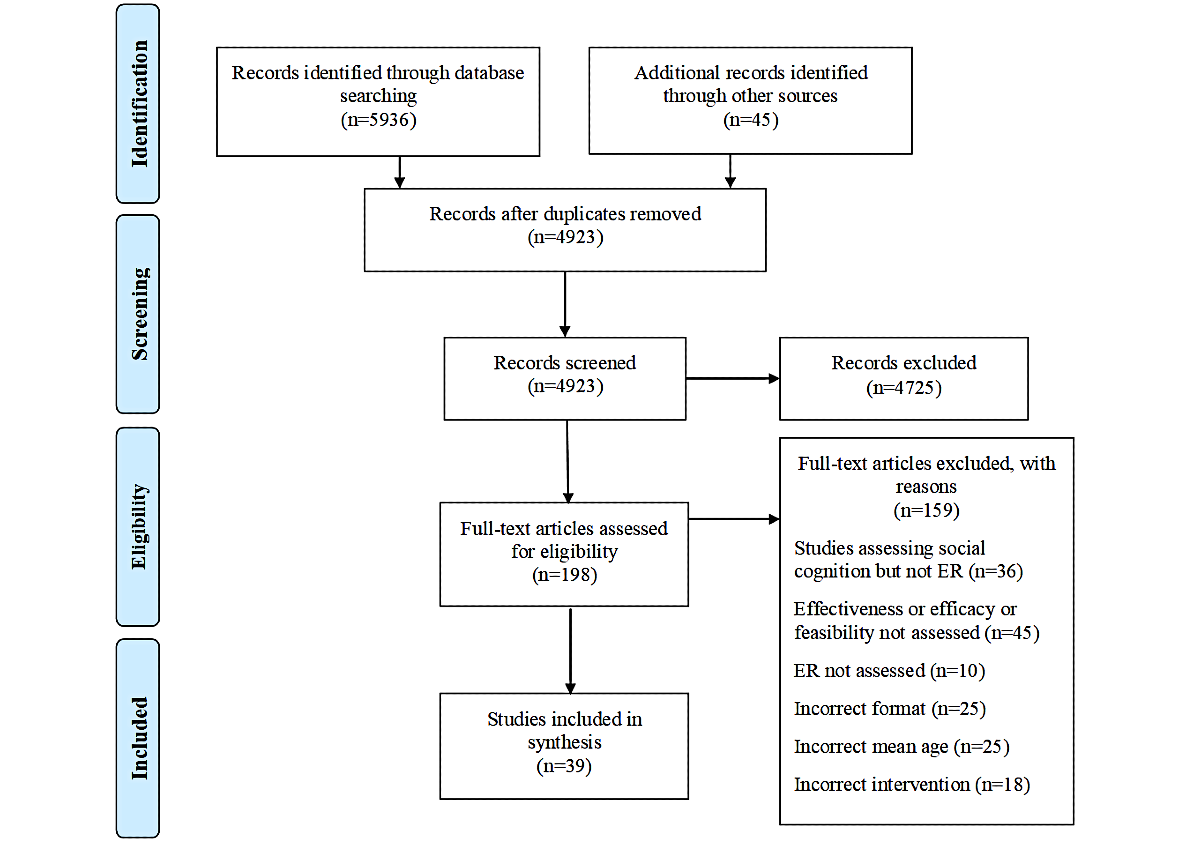
PRISMA (Preferred Reporting Items for Systematic Reviews and Meta-Analyses) flowchart for study inclusion (adapted from Moher et al [111], which is published under Creative Commons Attribution 4.0 International License). ER: emotion regulation.

The use of the inclusion and exclusion criteria as previously defined resulted in 39 studies being included in the systematic review and meta-analysis [112] (Figure 1).

### Description of Study Clustering

**Figure 2:**
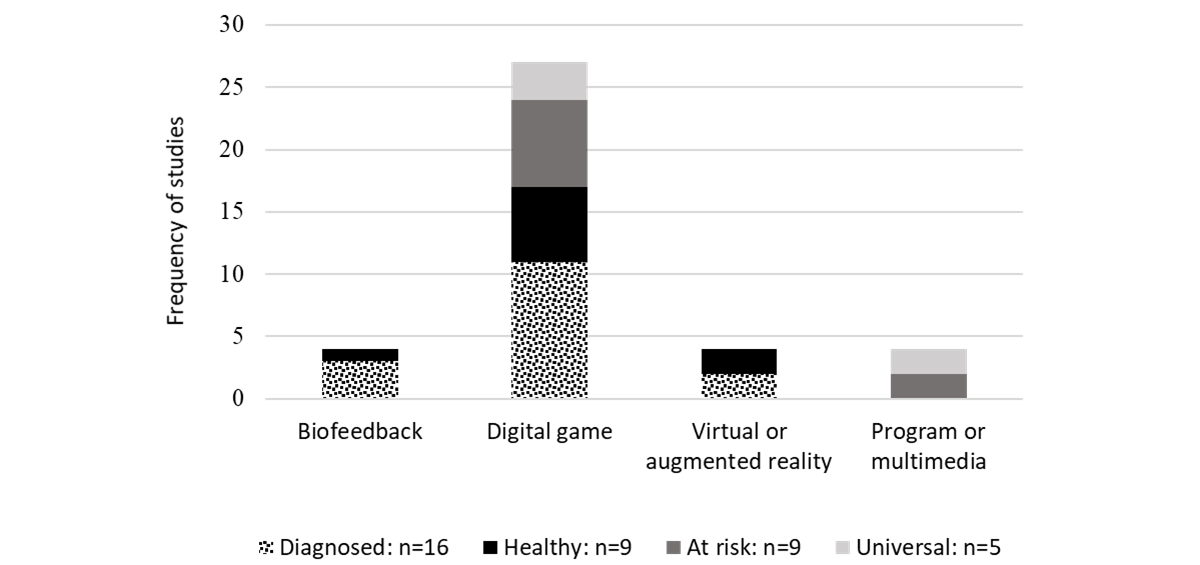
Study clustering findings with population characteristics. Of the 39 studies, 1 (3%) reported results for both populations who had received a diagnosis and healthy populations. The totals were calculated based on the main target population.

As shown in Figure 2, most (27/39, 69%) of the studies assessed digital game interventions in children and early adolescents who had received a diagnosis.

### Study Characteristics

Multimedia Appendix 4 [45-83] contains the characteristics of all included studies. The 39 studies had sample sizes ranging from 2 to 1645. Participants were aged 5 to 17 years, with a mean age, where reported, of 8 to 14 years. Studies provided data related to effectiveness (4/39, 10%); effectiveness and feasibility (11/39, 28%); effectiveness and acceptability (2/39, 5%); effectiveness, feasibility, and acceptability (9/39, 25%); efficacy (5/39, 13%); efficacy and feasibility (6/39, 15%); and efficacy, feasibility, and acceptability (2/39, 5%). Of the 17 studies that targeted children and early adolescents who had received a diagnosis, most (n=9, 53%) targeted autism spectrum disorders (ASDs). Of the 8 studies that targeted samples classified as *at risk*, half (n=4, 50%) targeted elevated anxiety with digital games. Studies were conducted in Australia (9/39, 23%), Spain (8/39, 21%), The Netherlands (6/39, 15%), the United States (6/39, 15%), Hong Kong (3/39, 8%), Romania (3/39, 8%), Wales (1/39, 3%), Nepal (1/39, 3%), Belgium (1/39, 3%), and Germany (1/39, 3%). Differentiation between effectiveness and efficacy highly depends on study design and available resources; indeed, effectiveness reflects real-life conditions. Hence, throughout the reporting of the results, we use the term *efficacy* for simplicity.

In total, 11 studies were eligible for meta-analyses. These comprised 2476 participants (n=1248, 50.4%, in intervention conditions and n=1228, 49.6%, in control conditions). Sample sizes ranged from 20 to 1645. Most of the studies targeted samples classified as at risk or diagnosed (8/11, 73%) and were digital games (9/11, 82%). Of the 9 digital games, 4 (44%) targeted children or early adolescents at risk of anxiety (n=448); 3 (33%) targeted those diagnosed with posttraumatic stress disorder, anxiety with and without comorbid intellectual disability, and ASD with elevated anxiety (n=178); and 2 (22%) targeted healthy early adolescents (n=185). Of the 11 studies, 1 (9%) biofeedback study targeted youth (n=20) diagnosed with anorexia nervosa, whereas 1 (9%) program and multimedia study targeted a universal sample (n=1645). No studies in the virtual and augmented reality cluster were included. Regarding comparisons, the digital game cluster compared the intervention with an active control (n=2), active control with (n=1) and without (n=2) treatment as usual, active control with separate wait-list (n=2), and treatment as usual with (n=1) and without (n=1) wait-list. The biofeedback study compared the intervention with treatment as usual. The program and multimedia study compared the intervention with a web-based neuroscience program. Of the 5 studies that permitted continuance of usual treatment, 3 (60%) were in the digital game cluster.

### Intervention Characteristic Summary

Of the 39 included studies, 22 (56%) clearly stated that they incorporated additional support, monitoring, or nondigital delivery. Most (3/4, 75%) of the program and multimedia studies incorporated class sessions and homework. Half (4/8, 50%) of the virtual and augmented reality studies as well as biofeedback studies and 56% (15/27) of the digital game studies incorporated nondigital delivery, additional support, or monitoring. Only the study by Wijnhoven et al [45] was included in the meta-analytic component. In total, 44% (17/39) of the studies trained other skills as well as emotion regulation. These were mostly (9/17, 53%) social skills and social cognition. A key pedagogical and therapeutic theme across all interventions was explicit emotion regulation strategy learning through digital characters or a face-to-face facilitator, with practice in a relevant and engaging but safe environment. Refer to Multimedia Appendix 5 for descriptions of all the interventions.

### Risk of Bias

**Figure 3:**
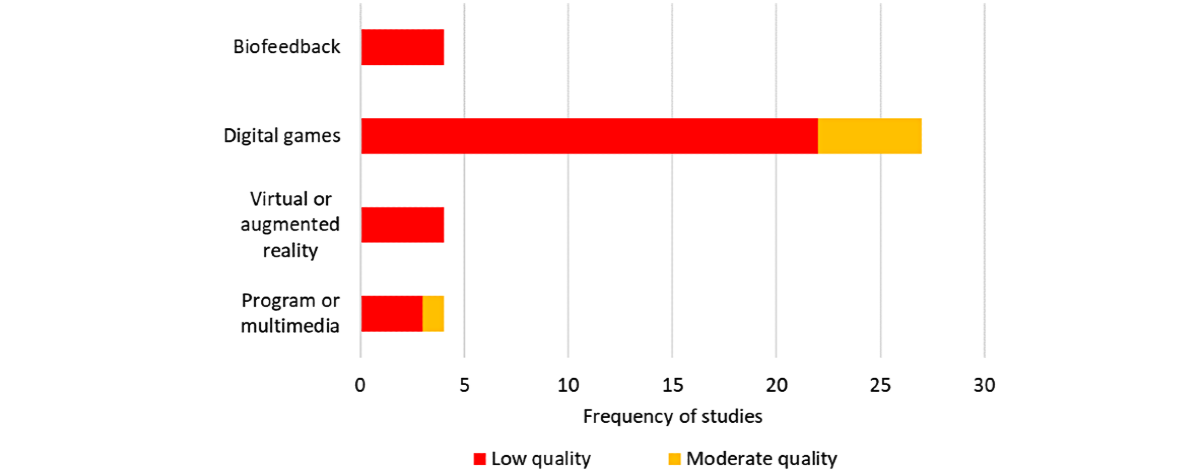
Review authors’ judgments regarding overall study quality in the intervention clusters.

As demonstrated in Figure 3, most (33/39, 85%) of the studies were judged by reviewers as low quality. In total, 15% (6/39) of the studies, all of which were included in the meta-analytic component, gained moderate quality ratings. Although most (17/39, 44%) of the studies targeted diagnosed populations, reviewers judged all these studies as low quality. The highest proportion of moderate quality ratings was in the digital game cluster in populations classified as at risk. Overall, the distribution of risk-of-bias scores ranged from 7 to 20 out of 26, with higher scores indicating higher quality. The interrater reliability was substantial (Cohen *κ*=0.75) [113]. Refer to Multimedia Appendix 6 for a detailed summary of the risk-of-bias findings.

### Meta-analysis

#### Emotion Experience

**Figure 4:**
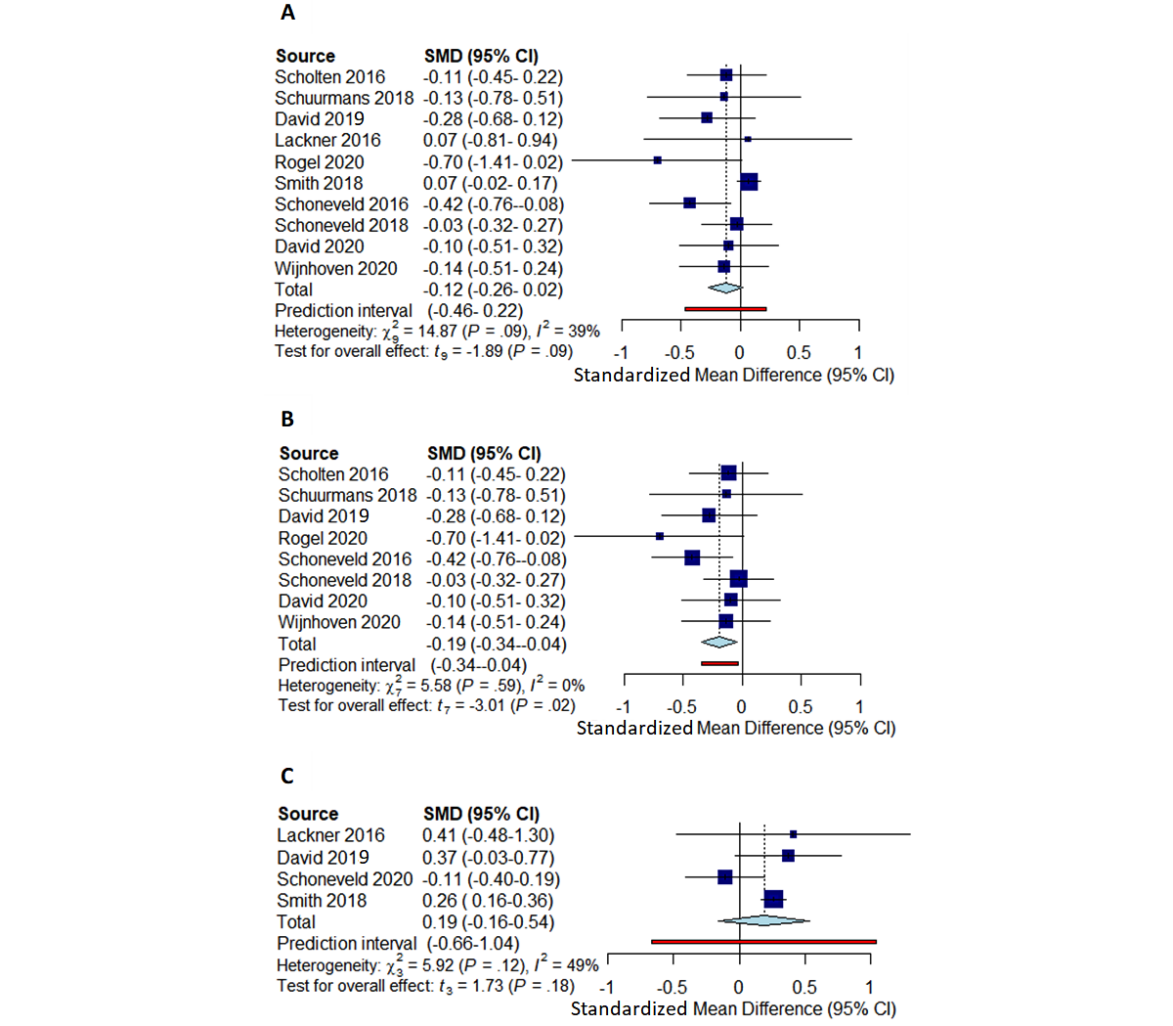
Meta-analytic forest plots (random effects model, Hedges *g*, restricted maximum likelihood tau-squared): (A) Emotion experience. (B) Emotion experience—digital game studies only. (C) Emotion regulation.

Of the 39 included studies, 10 (26%) assessed group differences in emotion experience with self-report. Of these 10 studies, 9 (90%) revealed effect sizes in favor of the intervention, with less negative (*k*=8) or more positive (*k*=1) emotion experience effects in the intervention group. However, of these 10 studies, only 1 (10%; digital game) revealed a significant effect (Table 1). This study targeted children at risk of anxiety. Only the study by Lackner et al [68] revealed an effect in the unexpected direction in which negative emotion experience was greater in the intervention group than in the control group after the intervention. This related to the only biofeedback study included in the meta-analysis, with the smallest sample size (n=20; although the study also reported one of the lowest dropout rates of 9%). The very small pooled effect was nonsignificant (*k*=10; Hedges g=–0.12, 95% CI –0.26 to 0.02; *P*=.09; Figure 4A). Tau-squared was low (*τ*^2^=0.0176) indicating little variation among the studies. Yet, the I^2^ value of 39.5% indicated low to moderate heterogeneity, and the somewhat broad prediction interval (–0.46 to 0.22) suggests that the very small observed pooled effect largely on negative emotion experience through emotion regulation digital interventions is not robust in every context.

Given the potential impact of the type of digital intervention on the pooled effect and heterogeneity outcomes, the emotion experience meta-analysis was conducted again with only the digital games studies (n=9). All the digital game studies assessed negative emotion experience outcomes. The forest plot reveals a small negative pooled effect (Figure 4B). This was significant (*k*=8; Hedges g=–0.19, 95% CI –0.34 to –0.04; *P*=.02). Tau-squared was 0, indicating that variation in effect sizes among the studies was caused by sampling error rather than heterogeneity. The *I*^2^ value of 0% corroborated this, and the narrow prediction interval (Hedges g=–0.34 to –0.04) suggests that the small observed pooled negative emotion experience effect through emotion regulation digital game interventions is robust across different contexts.

**Table 1:** Emotion experience and emotion regulation meta-analytic outcomes. Refer to Multimedia Appendix 7 [45-83] for measure details^a^.

Study, year [reference number]; intervention	Hedges g (95% CI)		Sample size, n	Control	Measures and risk of bias			Dropout rate, %		Other skill or support
	Emotion regulation	Emotion experience			Emotion regulation	Emotion experience				
Lackner et al^b^, 2016 [68]; biofeedback	0.41 (–0.48 to 1.3)	0.07 (–0.81 to 0.94)	20	TAU^c^	ECQ R^d,e^	BSI A^f,e^	9		No	
Scholten et al^g^, 2016 [73]; digital game	—^h^	–0.11 (–0.45 to 0.22)	138	Active	—	SCAS-C^i,e^	8.7 (ITT^j^)		No	
Schuurmans et al^b^, 2018 [75]; digital game	—	–0.13 (–0.78 to 0.51)	37	TAU	—	SCAS-C^e^	34 (ITT)		No	
David et al, 2019 [58]; digital game	0.37 (–0.03 to 0.77)	–0.28 (–0.68 to 0.12)	96	Active	ERICA C^k,e^	SDQ-C E^l,e^	7		No	
Rogel et al^b^, 2020 [71]; digital game	—	–0.70 (–1.41 to 0.02)	32	TAU-WL^m^	—	TSC A^n,e^	22 (ITT)		Executive function	
Schoneveld et al^g^, 2016 [46]; digital game	—	–0.42 (–0.76 to –0.08)^o^	136	Active	—	SCAS-C^e^	25.7 (ITT)		No	
Schoneveld et al^g,p^, 2018 [47]; digital game	—	–0.03 (–0.32 to 0.27)	174	Active	—	SCAS-C^e^	12 (ITT)		No	
David et al, 2020 [59]; digital game	—	–0.10 (–0.51 to 0.32)	89	Active	—	PoAD A^q,r^	18.8		No	
Wijnhoven et al^b^, 2020 [45]; digital game	—	–0.14 (–0.51 to 0.24)	109	Active	—	SCAS-C^r^	32		Therapist	
Schoneveld et al^g,p^, 2020 [74]; digital game	–0.11 (–0.4 to 0.19)	—	174	Active	SEQ SE^s,e^	—	12 (ITT)		No	
Smith et al^b^, 2018 [77]; program	0.26 (0.16 to 0.36)^t^	0.07 (–0.02 to 0.17)	1645	Active	ATES^u,r^	EWS^v,r^	0 reported		Adaptive attitude toward emotion regulation	

^a^Pooled Hedges g (random effects model, restricted maximum likelihood tau-squared): emotion regulation: Hedges g=0.19 (95% CI –0.16 to 0.54); emotion experience: Hedges g=–0.12 (95% CI –0.26 to 0.02), game only Hedges g=–0.19 (95% CI –0.34 to –0.04).

^b^Continuance of existing treatment permitted.

^c^TAU: Treatment as usual.

^d^ECQR: Emotional Competence Questionnaire, Regulating and Controlling Own Emotions subscale.

^e^Low risk of bias.

^f^BSIA: Brief Symptom Inventory, shortened from Symptom Checklist-90-Revised, Anxiety subscale.

^g^Continuance of existing treatment not permitted.

^h^Not available.

^i^SCAS-C: Spence Children’s Anxiety Scale.

^j^ITT: intention to treat used.

^k^ERICA C: Emotion Regulation Index for Children and Adolescents, Control subscale.

^l^SDQ-CE: Strengths and Difficulties Questionnaire–Child Version, Emotional Symptoms subscale.

^m^TAU-WL: Treatment as usual–waitlist.

^n^TSCA: Trauma Symptom Checklist for Young Children, Anxiety scale.

^o^Significant at *P*<.05.

^p^Noninferiority: no significant between-group differences expected.

^q^POAD A: Profile of Affective Distress, Concern and Anxiety subscale.

^r^High risk of bias.

^s^SEQ SE: Self-Efficacy Questionnaire for Children, Emotion Self-Efficacy scale.

^t^Significant at *P*<.01.

^u^ATES: Adaptive Theories of Emotions Scale.

^v^EWS: Emotional Well-Being in School Scale.

#### Emotion Regulation

Of the 11 studies included in the meta-analysis, 4 (36%) assessed group differences in emotion regulation with self-report. Only the study by Smith et al [77] (program) revealed a significant effect (Table 1). The non-inferiority study by Schoneveld et al [74] revealed an effect in favor of the control group. Of note is the biofeedback study by Lackner et al [68] in which the control group improved compared with the intervention group; yet, because the intervention group’s baseline mean was greater than that of the control group, the observed effect seems to be in favor of the intervention group. The pooled effect was nonsignificant (*k*=4; Hedges g=0.19, 95% CI –0.16 to 0.54; *P*=.18; Figure 4C). Tau-squared was low (*τ*^2^=0.0274), suggesting little variation among the studies. However, the *I*^2^ value of 49.3% indicated near-moderate heterogeneity, and the extremely broad prediction interval (–0.66 to 1.04) suggests that the nonsignificant small observed pooled effect on emotion regulation through emotion regulation digital interventions is not robust.

#### Outliers and Influential Cases

Outlier analysis did not detect any extreme effect sizes for the emotion experience or emotion regulation meta-analyses.

In the meta-analysis on emotion experience outcomes in digital games (significant), no studies were identified as extreme cases using the influential Viechtbauer and Cheung study threshold [106]; yet, visual inspection of the influence analysis subplots suggested that the studies by Schoneveld et al [46] and Schoneveld et al [47], both of which trained emotion regulation with an electroencephalogram (EEG) neurofeedback–based anxiety-induction digital game, presented extreme values. The Baujat plot corroborated this, indicating that these studies were highly influential in heterogeneity and pooled effect size. These studies also measured efficacy expectancy before the intervention and reported null between-group differences. Refer to Multimedia Appendix 8 [46,74,77,106] for a detailed description of the influence analyses.

In summary, the meta-analytic evidence suggests that only digital game interventions significantly reduced negative emotional experience in children and early adolescents with a small effect, and this may be robust across different contexts; yet, there is no evidence for improvements in self-reported emotion regulation abilities through digital intervention.

### Publication Bias

**Figure 5:**
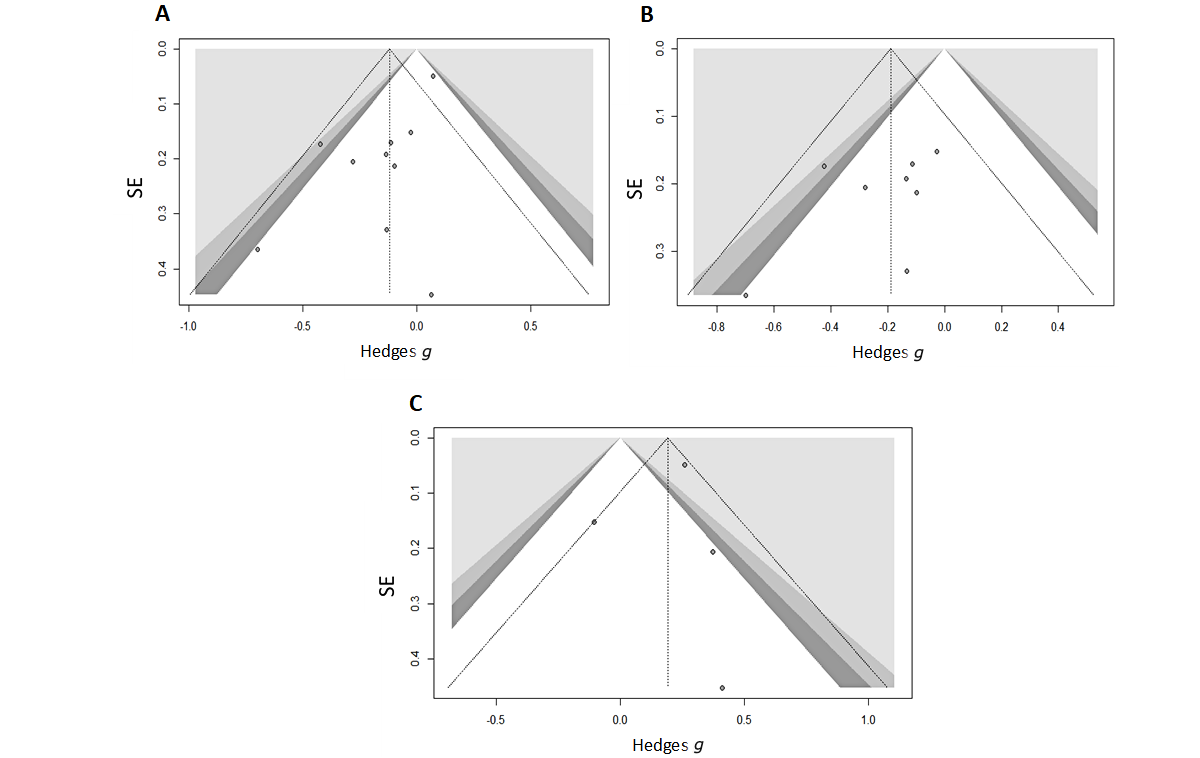
Meta-analytic contour-enhanced funnel plots between the SE and Hedges *g*. (A) Emotion experience. (B) Emotion experience—digital game studies only. (C) Emotion regulation. Light gray shading: *P*<.01; gray shading: *P*<.025; and dark gray shading: *P*<.05. No shading: nonsignificant (*P*<.05).

Visual inspection of the contour-enhanced funnel plots (Figure 5) indicated some asymmetry. Importantly, there was only 1 significant effect size in each funnel plot. This suggests that asymmetry may have been due largely to factors other than publication bias (eg, variations in study quality and methodology).

The Egger test of the intercept was nonsignificant for both the emotion regulation (*k*=4; regression intercept –0.409, 95% CI –3 to 2.19; *P*=.79) and emotion experience digital game–only meta-analyses (*k*=8; regression intercept –1.514, 95% CI –3.87 to 0.84; *P*=0.25). However, it was significant for the emotion experience meta-analyses that included all relevant studies (*k*=10; regression intercept –1.462, 95% CI –2.36 to –0.57; *P*=.01). Hence, there was substantial asymmetry within this funnel plot potentially because of variations in study quality and methodology.

A trim-and-fill analysis was conducted on the significant emotion experience effect (refer to Multimedia Appendix 9 for the associated funnel plot). The 5 added effects were larger in magnitude, and the pooled effect was smaller and remained nonsignificant (*k*=15; Hedges *g*=–0.019, 95% CI –0.16 to 0.2; *P*=.82). Tau-squared was moderate (*τ*^2^=0.05), indicating variation among the studies. I^2^ was 56%, indicating moderate heterogeneity.

In summary, the small, significant impact of *digital games* on negative emotional experience in children and early adolescents was likely *not* overestimated because of either publication bias or variations in study quality and methodology.

### Efficacy

#### Overview

Multimedia Appendix 10 [48-57,60-67,69,70,72,76,78-83] contains the within-intervention group pre- to postintervention efficacy summaries of emotion regulation, emotion experience, and physiological regulation domains from all studies not included in the meta-analyses (28/39, 72%). Where available, follow-up data are also provided. Studies were nonrandomized or noncontrolled or judged to not be adequately resistant to biases and of variable methodological design. Therefore, the synthesis assessments described in the following sections should be interpreted accordingly. Where it was not possible to synthesize before-and-after efficacy data (eg, single-session experiments, postintervention interviews, and field notes), data were synthesized in line with the measures and design from which they were borne.

#### Biofeedback Studies

Of the 3 biofeedback studies that provided efficacy data, only 1 (33%) used measures that were judged as low risk of bias. All 3 studies provided data on physiological regulation. Children and early adolescents significantly altered their physiology as directed by the intervention in heart rate variability (HRV)–EEG and functional magnetic resonance imaging (fMRI) biofeedback-neurofeedback and neurofeedback interventions. Emotion regulation was assessed in 67% (2/3) of these studies: emotion regulation correlated with increased emotion regulation network activation in the fMRI neurofeedback study, whereas emotion regulation improved significantly with a large effect in HRV biofeedback but not in combined HRV-EEG biofeedback-neurofeedback in the second study. This study also assessed negative emotion experience—emotional lability and negativity decreased significantly with a large effect. Anxiety reductions were nonsignificant.

#### Digital Game Studies

Of the 27 digital game studies, 18 (67%) provided efficacy data. Of these 18 studies, 3 (17%) assessed the success of frustration or joy emotion induction within a virtual reality–enabled emotion regulation game across different mediating devices. Frustration increased significantly after the frustrating game in 33% (1/3) of the studies but not when mediated by a camera device. Increases in joy after the joyful game were nonsignificant across all device types in 33% (1/3) of the studies.

The strongest evidence for positive change brought about by digital games was the reduction of negative emotion experience (anxiety). Of the 18 studies, 7 (39%) measured this; of these 7 studies, 3 (43%) were statistically significant with small to large effects.

Of the 18 studies, 8 (44%) measured emotion regulation, which largely improved. Where pre-post statistical information was available (5/8, 63%), improvements were significant, with medium to large effects. However, the significant findings reported on the same game.

Of the 18 studies, 5 (28%) assessed physiological regulation. In total, 20% (1/5) of the studies reported significant reductions in heart rate and 20% (1/5) reported nonsignificant reductions in heart rate.

#### Virtual and Augmented Reality

Of the 4 virtual and augmented reality studies, 4 (100%) provided efficacy data, largely with measures judged as low risk of bias. Most (3/4, 75%) of the studies only measured emotion regulation. Individual exposure but not group exposure to immersive virtual reality emotion and social skill practice was linked to significant improvements in emotion regulation in a sample with high-functioning ASD, with a small effect (from 2/4, 50%, studies).

#### Program and Multimedia

Of the 4 program and multimedia studies, 3 (75%) provided efficacy data, all of which assessed 1 multimedia modular program. Intensity of emotions was assessed in 67% (2/3) of these studies—intensity of negative emotions only decreased significantly in 50% (1/2) of these studies, with a small effect. Intensity of positive emotions decreased significantly in both studies, with small to large effects.

#### Summary of Efficacy Data

The most consistent evidence comes from digital game interventions in the reduction of negative emotion experience. A note of caution is recommended when interpreting these findings owing to the varied methodology, high risk of bias, and overall low quality of the included studies. Furthermore, the evidence base for the impact of digital interventions on physiological indices of emotion regulation is much smaller and less consistent.

### Feasibility

Multimedia Appendix 11 [45-51, 53, 54, 56, 58, 59, 65, 66, 68-79, 82, 83] contains the feasibility summaries from the included studies. Feasibility data were provided for 72% (28/39) of the studies. All studies that provided feasibility data used measures judged as high risk of reporting bias.

Of the 28 studies, 18 (64%) digital game studies provided feasibility data for various aspects of feasibility, including engagement, implementation, adherence, expectations, and transference to real life. Where the dropout rate was particularly high (>30%), studies targeted samples who had received a diagnosis and the dropouts were largely attributed to personal or family issues. Most feasibility issues were in early-stage small studies (3/18, 17%) in which interventions were prototypes not previously evaluated or were delivered by individuals inexperienced in the intervention technology.

All (4/4, 100%) the virtual and augmented reality studies provided feasibility data encompassing engagement, implementation, and transference to real life. Of these 4 studies, 2 (50%) reported dropout rates, and these were very low.

Of the 4 program and multimedia studies, 3 (75%) provided feasibility data encompassing implementation and engagement. Of these 3 studies, 2 (67%) reported dropout rates, and these were very low.

In summary, most feasibility issues were in early-stage interventions targeting samples who had received a diagnosis. Digital game interventions that incorporated biofeedback provided the most evidence for transference of learned emotion regulation skills to real life. However, digital games also presented the highest dropout rate, and all measures across all clusters were judged as high risk of reporting bias.

### Acceptability

Multimedia Appendix 12 [46-49,53,54,65,67,69,72,75,80,82] contains the acceptability summaries from the included studies. Acceptability data were provided for 33% (13/39) of the studies. The biofeedback cluster did not contain acceptability data.

Of the 9 digital game studies that measured acceptability, 6 (67%) reported moderate to highly positive results for at least one aspect of acceptability, including likability, flow, usability, helpfulness, difficulty, appeal, usefulness, and relevance. The only study that reported mainly negative acceptability findings highlighted a link between guided imagery, visualization, and deep breathing games being too difficult or easy and poor likability in children diagnosed with ASD.

Of the 4 virtual and augmented reality studies, 2 (50%) that evaluated acceptability in 2 interventions reported mainly positive findings for fun, educational impact, likability, motivation impact, and experienced happiness. The only study across all clusters that used a measure judged as low risk of reporting bias assessed an outdoor augmented reality quest (which involved meeting other players). Importantly, it was viewed as potentially dangerous, although the authors did not elucidate exactly to what this *danger* pertained.

Of the 4 program and multimedia studies, 2 (50%) that evaluated acceptability in a school-based program reported high likability and a moderate educational impact.

In summary, emotion regulation digital interventions were largely acceptable to children and early adolescents, as well as other key stakeholders. However, of the 20 measures, 19 (95%) were judged as high risk of reporting bias. Negative acceptability findings were mainly in small early-stage digital game interventions targeting samples who had received a diagnosis.

## Discussion

### Summary

This systematic review and meta-analysis aimed to evaluate current digital interventions that train emotion regulation in children and early adolescents published in peer-reviewed articles up to July 2020. In summary, digital games were the most prevalent intervention type: 69% (27/39) of the studies evaluated digital games. Digital games decreased negative emotional experience with a small significant effect, mainly in samples at risk of anxiety. In addition, digital interventions improved emotion regulation; yet, this effect was nonsignificant. Furthermore, acceptability was strong across all intervention types and samples, and most feasibility-related problems were in samples who had received a diagnosis. In the following sections, we discuss the key findings and provide recommendations for the field’s progression.

### Efficacy

Examined through meta-analysis and systematic review, digital games provided evidence for a significant reduction in negative emotional experience with a small effect, largely in samples at risk of anxiety, using validated and reliable outcome measures. This suggests that digital games are the most advanced and efficacious digital interventions for training emotion regulation in children and early adolescents. This important finding may be partly explained with cognitive load theory, which postulates that limited novel information can be processed at once in working memory [114]. Indeed, to optimize learning in a digital environment, balance must be sought between presenting information in a manner that meets an individual’s cognitive needs, yet with sufficient complex information to facilitate understanding of the given topic, and learning must be active to enhance the development of cognitive schemas [114,115]. Such optimization may be achieved with certain pedagogical techniques. For example, pacing serves to decrease cognitive load on working memory by relying on the user or system to control information presentation (eg, by pausing material delivery or going back to look at previous material) [115]. In line with these digital pedagogical principles, the included digital game studies largely presented learning tasks that focused on different emotion regulation strategies within separate parts of the game, with gradual user-led increases in difficulty and complexity, and a simple user-friendly interface, with animated characters that provided information about different emotion regulation strategy elements and in-game support.

In combination with digital game design methods that optimize cognitive flow [116], feelings of autonomy [117,118], and fun [119], digital game training may have increased motivation and engagement, which are recognized barriers to efficacy in digital interventions in children and early adolescents [40].

Neurofeedback may also be key to this finding; meta-analytic influence analysis indicated that the digital game studies that incorporated EEG neurofeedback (2/27, 7%) drove the small significant pooled effect. This is in line with the embodied emotion regulation framework [120], which proposes a distinction between cognitively based top-down (cognitive labeling, mindful detachment, meta-awareness, and cognitive reprisal) and affect-driven bottom-up (sensory perception and interceptive proprioception) emotion regulation strategies and argues that they work together as part of an integrated emotion regulation system. Hence, it is possible that these interventions successfully addressed both top-down and bottom-up strategies, which increased efficacy. In addition, the real-time visual neurofeedback may have further increased immersion within the digital game and, subsequently, engagement [40]. However, neurofeedback information provided to players was collected using non–research-grade EEG equipment, and double blinding was not incorporated. In this context, the role of placebo effects on the apparent impact of neurofeedback on clinical symptomatology must be considered. This was discussed by 3 studies [121-123] in line with prior clinical neurofeedback research in which diligent methodological rigor is not evident; yet, significant intervention effects are routinely reported. The emotion regulation digital intervention field should address concerns around potential placebo effects in neurofeedback through the application of methodological rigor, including double-blind, placebo-controlled trials [122].

When planning placebo-controlled trials it is important to consider that expectations around intervention effects may influence placebo effects; yet, such expectations are rarely measured in light of this [124]. The higher-quality digital game studies (2/27, 7%) in this review that drove the emotion experience findings measured intervention expectation at baseline and reported null between-group effects. However, earlier-stage studies, not included in the meta-analytic component, did not. As the field progresses, intrinsic motivation must be harnessed in double-blind, placebo-controlled trials, with expectancy measured at baseline, particularly in studies that incorporate neurofeedback components. In addition, although portability and ease of use drive the use of non–research-grade EEG equipment, it is argued that such issues must be balanced against the impact on the credibility of the tool. That is, if digital emotion regulation training in children and early adolescents relies on suboptimal technology, are we really driving the field forward?

There was limited measurement of emotion regulation across all included studies; hence, the available emotion regulation efficacy findings must be interpreted with caution. The lack of focus on emotion regulation may be due to the included studies focusing somewhat on children and early adolescents at risk of *anxiety* and the concurrent training of *social cognition* and *social skill* difficulties; hence, these constructs were the key outcomes. In addition, there are limited psychometrically sound emotion regulation measures for children and adolescents, despite increasing awareness of the importance of its adaptive development [125]. To advance the field, there is a requirement for researchers to create and validate emotion regulation measures for diverse child and early adolescent samples, and digital intervention studies should objectively assess improvements in emotion regulation ability after the intervention and at follow-up.

Considering emotion regulation *knowledge*, medium to large significant improvements were observed in digital game studies that also applied additional therapeutic support, parental guidance, or targeted social cognitive skills, particularly in samples with ASD. Indeed, research has highlighted associations between brain regions implicated in cognitive emotion regulation and social cognition in youth [126] and the requirement of perspective taking [127] and abundant semantic representations [128] for successful alternative representations of emotion-inducing stimuli (ie, cognitive reappraisal). Furthermore, the integration of caregivers in interventions for samples with ASD may boost the generalizability of learned skills [129] and increase engagement with the intervention [130]. Hence, the inclusion of social cognition training as well as caregiver support may have positively influenced the emotion regulation improvements observed in these studies. Therefore, it may be beneficial to include social cognitive training and parental support within emotion regulation digital interventions that target samples with ASD because this may enhance their efficacy. However, as emotion regulation knowledge improvement was only assessed in a small number of lower-quality studies, we recommend that caution must be taken when interpreting such findings.

### Feasibility

Small early-stage digital game studies that targeted ASD, attention-deficit/hyperactivity disorder, and samples with undefined emotional disorders identified several important feasibility issues linked to generalization, implementation, technical issues, and physiological and emotional symptoms. Interindividual variability and related intervention difficulties are common in samples with neurodevelopmental disorders [131]. Hence, greater feasibility difficulties in such samples are expected. Furthermore, had feasibility issues not been picked up at this early stage of evaluation, full-scale evaluation may have yielded less favorable findings. A program and multimedia intervention assessed in slightly larger studies (2/39, 5%) found that the content was too complex for children at risk of exclusion or suspension from school, and postintervention reductions in the intensity of emotions were variable. Had the ability of the target sample to understand intervention content been checked at an early intervention development stage, efficacy outcomes might have been more consistently positive because the key messages would have been better understood. The relative importance of early-stage studies is emphasized through the consideration of the Medical Research Council’s guidelines for complex intervention development [132]. Here, the impact of contextual factors on intervention success is highlighted and has recently been discussed further in a digital intervention context [40]—it is advised that iterative feasibility assessments that examine the issues revealed throughout intervention development are key to understanding contextual factors.

A further key finding was the higher dropout rate in samples who had received a diagnosis, especially in the digital game cluster. It is possible that because digital games made up a high proportion of the included studies, they also presented the most realistic picture of dropouts in digital interventions for emotion regulation. Moreover, because digital games were largely evaluated in terms of their effectiveness in real-world settings (eg, school, home, and inpatient care) this may have affected adherence and, subsequently, dropouts. Certainly, adherence to digital interventions outside of research settings in children and early adolescents is an extant key issue [12,133]. Involving the population in the design process who will ultimately use the digital intervention may lead to the iterative development of tools that are feasible and address the high dropout rate [40]. Methods to involve youth in digital intervention development may be optimized to increase engagement [40]. These include using progress bars, animations, and multiple platforms in web-based questionnaires; usability *think-aloud* protocols instead of standard interviews (observed and/or interviewed simultaneously while using the intervention); clear rules and use of materials (eg, screens and devices) in focus groups; and principles applied to focus groups with *wall storms* (sticky notes on a wall) and *word clouds* (grouping of key words) in participative workshops [40].

As digital games that incorporated biofeedback provided the greatest evidence for generalizability of learned emotion regulation skills, this suggests that biofeedback-based digital games may be the most appropriate emotion regulation digital intervention for transference to real life; yet, there is no extant empirical research to support this. Objective assessment of generalization was only conducted in biofeedback-based digital games in samples who had received a diagnosis *(psychiatric and neurodevelopmental disorders)*; hence, this finding may simply be an artifact of the relative prominence of biofeedback-based digital game interventions and inadequate measurement of generalization in the other included studies, although it is important to consider the significance of the specific emotion regulation strategies—deep breathing and cognitive emotion regulation—that seemed to demonstrate the greatest real-life generalizability in children and early adolescents who had received a diagnosis. fMRI-based and self-report–based evidence in adult populations suggests that cognitive reappraisal may be linked to future rather than immediate emotion regulation success in reducing negative emotion (ie, when emotion-inducing stimuli are re-encountered at a later date) [134]. This suggests that the real-life relevance of content within digital cognitive emotion regulation training may be particularly important such that it should clearly relate to the target samples’ *real-life experiences and difficulties* to promote future use of learned strategies. In addition, higher cognitive reappraisal frequency is linked to reduced risk for psychiatric symptomatology [135], optimal academic attainment [136], social outcomes [137], and psychological well-being [138].

Researchers should collaborate with key stakeholders to create highly relevant and engaging intervention components of appropriate complexity to produce improvement in indices of emotion regulation with real-world generalizability [40,139]. Importantly, generalizability should be consistently assessed to determine the emotion regulation digital interventions that are most appropriate across different child and early adolescent samples.

### Acceptability

The only study that provided solely negative acceptability data assessed emotion regulation mini-games in an early-stage small evaluation. Here, poor likability was linked to unsuitable difficulty for individuals’ needs in a sample with high-functioning ASD. As mentioned previously, a key factor in the presentation of ASD and associated interventions is interindividual variability and related intervention difficulties [131]. Digital technologies allow for greater person-centered training through the involvement of caregivers and the ability to engage with the intervention at home [131]. However, if the caregiver is not able to quickly and easily adjust the difficulty of the intervention or if it is not programmed to adapt dynamically, as also reported in the highlighted study, such caregiver involvement may be in vain [140]. Hence, it is recommended that emotion regulation digital games, especially those designed for children and early adolescents with neurodevelopmental disorders, should incorporate game mechanics that adapt dynamically to an individual’s needs, permitting increases and decreases in difficulty as required in real time. For this to be successful, interdisciplinary collaboration is required at all stages of conceptualization, specification, and programming [141]. Specific collaborators may include psychologists, cognitive neuroscientists, educators, therapists, engineers, and, principally, the target of the intervention (ie, youth) [40,139,142].

A further notable finding was the importance of relevance in emotion regulation digital games. Specifically, in an immersive EEG-neurofeedback and anxiety-induction game set within a haunted mansion with ghosts, the experience of relevance to real life was significantly less than that in a group-based CBT comparator but not less than that in a nontherapeutic commercial game comparator. Hence, it may be important to include explicit training content in emotion regulation games that clearly relates to the target samples’ *real-life experiences and difficulties* and encourages children and early adolescents to practice learned skills in their daily lives, optimize acceptability, and encourage generalization, as practiced in traditional talking therapies (eg, CBT and dialectical behavior therapy) [20,22]. However, appeal and flow were also key to the experience of acceptability in emotion regulation digital games—the evidence suggests that the experience of these aspects of acceptability may be inferior in emotion regulation games compared with commercial games. Consequently, because relevance, appeal and flow may come into conflict in emotion regulation digital game acceptability, it is recommended that a balance between them should be struck to optimize acceptability. This requires iterative codevelopment at all stages of evaluation [40,139].

Of vital importance to any research activity is the safety of participants, both objectively and through their own subjective experience. Perhaps reflective of the limited acceptability evaluation yet great variability in the type of acceptability assessed in the included studies, only 3% (1/39) of the studies assessed feelings of safety—a commercial augmented reality outdoor-based quest (Pokémon GO) found that participants experienced high levels of perceived danger when engaging in the intervention. Although details were not reported, it is sensible to construe that this may be in relation to the potential for harm from strangers because of interaction with unknown players. Hollis et al [143] provided an overview of the extant cultural and political debate and related research concerning the role and impact of digital technology in the lives of youth. Describing it as a *triple-edged sword*, the authors stated that it fosters personal development and growth; may detect and address mental health issues; and yet could pose purported social, intellectual, and mental health risks. This debate is increasingly heightened because digital technology (and the means to access it) is more important than ever in supporting the educational and socioemotional needs of youth through the COVID-19 pandemic. Crucially, most social, intellectual, and mental health concerns around the impact of new digital technologies—largely driven by population as well as political and academic arenas—may be challenged through nuanced research examining the impact of digital technology on those using it [144].

### Limitations

Considering the included studies, it is necessary to interpret the significant meta-analytic effect on the reduction of emotional experience in digital games with caution because of potential placebo effects, as discussed previously. In addition, only postintervention results were presented in the majority (28/39, 72%) of studies. This limited the ability to assess whether immediate improvements persisted and for how long. Hence, it is recommended that follow-up assessments should be conducted in large-scale studies that assess efficacy. Moreover, systematic review findings and subsequent discussions should be interpreted with caution because of the high risk of bias exhibited within the outcome measures. Researchers should endeavor to use validated and reliable acceptability and feasibility measures, and where this is not possible (eg, when obtaining nuanced qualitative information in iterative development workshops, web-based activities, focus groups, or interviews), a clear acknowledgment and explanation of the implications of using measures that may bias the outcomes should be provided. Finally, evident in this review is the limited number of large-scale RCTs. To push the emotion regulation digital intervention field forward, a transformation of the ethics and review board application process is required [141]. Currently, funding review panels frequently require highly detailed study protocols, with little to no consideration for the flexibility that is necessitated in collaborative design [141]. Encouraging greater flexibility in emotion regulation digital intervention development and evaluation plans may permit a stronger research focus on vital acceptability and feasibility features and lead to the successful growth of this emerging field.

Although the findings discussed here reveal potential benefits of, and provide recommendations for, the rigorous progression of emotion regulation digital interventions in children and early adolescents, this systematic review and meta-analysis does include some limitations. The search strategy was broad, which may be seen as a strength at this early stage of the field’s progression because it is imperative to understand the breadth of factors that may be implicated in its advancement. By contrast, this increased the number of required focal points of the review, which may have reduced its specificity. Furthermore, the focus on childhood and early adolescence is a strength—it enabled a nuanced understanding of this important developmental period. However, the meta-analysis did not include informant-reported effects. Although this decision was made to ensure homogeneity of the selected effect sizes, it might have limited the understanding of the benefits of emotion regulation digital intervention.

In conclusion, this review provides an important first step in the progression of emotion regulation digital interventions by synthesizing efficacy, feasibility, and acceptability data, published from 2008 to 2020, with a focus on childhood to early adolescence. The most consistent evidence came from digital games in the reduction of negative emotions, principally in children at risk of anxiety. However, variable methodologies, lack of follow-up assessment, and high risk of bias, inclusive of potential placebo effects within statistically influential neurofeedback-based digital game studies, limit definitive conclusions that may be made regarding the efficacy of such interventions. Engaging iterative intervention codevelopment with the sample who will eventually use the digital intervention and properly adjusting the difficulty to the intervention target is vital in achieving optimal acceptability and, specifically, addressing concerns around engagement. Finally, large-scale studies that assess emotion regulation as a key outcome using valid and reliable measures are urgently required to assess the extent to which emotion regulation ability may be improved in different samples of children and early adolescents through digital technology.

## Appendix

Multimedia Appendix 1 Database search strings.

Multimedia Appendix 2 Master extraction table.

Multimedia Appendix 3 Risk-of-bias tool domains and definitions.

Multimedia Appendix 4 Intervention characteristic matrix summary.

Multimedia Appendix 5 Intervention characteristic summary.

Multimedia Appendix 6 Risk-of-bias result summary.

Multimedia Appendix 7 Study and measures matrix.

Multimedia Appendix 8 Meta-analysis influence analysis summary.

Multimedia Appendix 9 Meta-analysis trim-and-fill funnel plot.

Multimedia Appendix 10 Intervention efficacy matrix.

Multimedia Appendix 11 Intervention feasibility matrix.

Multimedia Appendix 12 Intervention acceptability matrix.

## Abbreviations

ASD: autism spectrum disorder
CBT: cognitive behavior therapy
EEG: electroencephalogram
fMRI: functional magnetic resonance imaging
HRV: heart rate variability
NHS: National Health Service
PRISMA: Preferred Reporting Items for Systematic Reviews and Meta-Analyses
RCT: randomized controlled trial
