# Supplementary material for: Digital Interventions for Emotion Regulation in Children and Early Adolescents: Systematic Review and Meta-analysis
Source: JMIR Serious Games. 2022 Aug 19;10(3):e31456. doi: 10.2196/31456 (PMC9440412; doi:10.2196/31456)
Supplement: Multimedia Appendix 1 [file games_v10i3e31456_app1.docx]

**Multimedia Appendix 1. Database search strings**

| Database | Search string |
| --- | --- |
| Web of Science | (child* OR adoles* OR teenage* OR youth)  AND  (intervention* OR placebo* OR train* OR program* OR teach OR prevent* OR therap* OR strateg* OR treat*)  AND  (digit* OR technolog* OR internet OR virtual OR VR OR online OR neurofeedback OR portal OR e-portal OR “digital portal” OR “web based portal” OR “online portal” OR e-platform OR “online platform” OR “web based platform” OR “digital platform” OR wearable OR biofeedback OR comput* OR gamification OR game* OR gaming OR videogame OR “artificial intelligence” OR AI OR “augmented reality” OR AR OR e-health OR web OR cyber OR multimedia OR remote* OR app OR application OR “mobile technology” OR “mobile device” OR “mobile platform” OR smartphone OR phone OR “handheld device” OR “handheld application” OR interactive OR “digital device”) AND  (“emotion* regulation” OR “emotion* management” OR “affect regulation” OR “self control” OR “self regulation” OR “emotion* control” OR “affective self control” OR "social cognition" OR "perspective taking" OR “metacognition” OR “mentalis*” OR “mentaliz*” OR “TOM” OR "theory of mind") |
| PsychINFO | child* OR adoles* OR teenage* OR youth  AND  intervention* OR placebo* OR train* OR program* OR teach OR prevent* OR therap* OR strateg* OR treat*  AND  digit* OR technolog* OR internet OR virtual OR VR OR online OR neurofeedback OR portal OR e-portal OR digital portal OR web based portal OR online portal OR e-platform OR online platform OR web based platform OR digital platform OR wearable OR biofeedback OR comput* OR gamification OR game* OR gaming OR videogame OR artificial intelligence OR AI OR augmented reality OR AR OR e-health OR web OR cyber OR multimedia OR remote* OR app OR application OR mobile technology OR mobile device OR mobile platform OR smartphone OR phone OR handheld device OR handheld application OR interactive OR digital device  AND  emotion* regulation OR emotion* management OR affect regulation OR self control OR self regulation OR emotion* control OR affective self control OR social cognition OR perspective taking OR metacognition OR mentalis* OR mentaliz* OR TOM OR theory of mind |
| Medline | child* OR adoles* OR teenage* OR youth  AND  intervention* OR placebo* OR train* OR program* OR teach OR prevent* OR therap* OR strateg* OR treat*  AND  digit* OR technolog* OR internet OR virtual OR VR OR online OR neurofeedback OR portal OR e-portal OR digital portal OR web based portal OR online portal OR e-platform OR online platform OR web based platform OR digital platform OR wearable OR biofeedback OR comput* OR gamification OR game* OR gaming OR videogame OR artificial intelligence OR AI OR augmented reality OR AR OR e-health OR web OR cyber OR multimedia OR remote* OR app OR application OR mobile technology OR mobile device OR mobile platform OR smartphone OR phone OR handheld device OR handheld application OR interactive OR digital device  AND  emotion* regulation OR emotion* management OR affect regulation OR self control OR self regulation OR emotion* control OR affective self control OR social cognition OR perspective taking OR metacognition OR mentalis* OR mentaliz* OR TOM OR theory of mind |
| EMBASE | child* OR adoles* OR teenage* OR youth  AND  intervention* OR placebo* OR train* OR program* OR teach OR prevent* OR therap* OR strateg* OR treat*  AND  digit* OR technolog* OR internet OR virtual OR VR OR online OR neurofeedback OR portal OR e-portal OR digital portal OR web based portal OR online portal OR e-platform OR online platform OR web based platform OR digital platform OR wearable OR biofeedback OR comput* OR gamification OR game* OR gaming OR videogame OR artificial intelligence OR AI OR augmented reality OR AR OR e-health OR web OR cyber OR multimedia OR remote* OR app OR application OR mobile technology OR mobile device OR mobile platform OR smartphone OR phone OR handheld device OR handheld application OR interactive OR digital device  AND  emotion* regulation OR emotion* management OR affect regulation OR self control OR self regulation OR emotion* control OR affective self control OR social cognition OR perspective taking OR metacognition OR mentalis* OR mentaliz* OR TOM OR theory of mind |
| Education Resources Information Centre | child* OR adoles* OR teenage* OR youth  AND  intervention* OR placebo* OR train* OR program* OR teach OR prevent* OR therap* OR strateg* OR treat*  AND  digit* OR technolog* OR internet OR virtual OR VR OR online OR neurofeedback OR portal OR e-portal OR digital portal OR web based portal OR online portal OR e-platform OR online platform OR web based platform OR digital platform OR wearable OR biofeedback OR comput* OR gamification OR game* OR gaming OR videogame OR artificial intelligence OR AI OR augmented reality OR AR OR e-health OR web OR cyber OR multimedia OR remote* OR app OR application OR mobile technology OR mobile device OR mobile platform OR smartphone OR phone OR handheld device OR handheld application OR interactive OR digital device  AND  emotion* regulation OR emotion* management OR affect regulation OR self control OR self regulation OR emotion* control OR affective self control OR social cognition OR perspective taking OR metacognition OR mentalis* OR mentaliz* OR TOM OR theory of mind |
| ACM Digital Library | child* OR adoles* OR teenage* OR youth  AND  intervention* OR placebo* OR train* OR program* OR teach OR prevent* OR therap* OR strateg* OR treat*  AND  digit* OR technolog* OR internet OR virtual OR VR OR online OR neurofeedback OR portal OR e-portal OR “digital portal” OR “web based portal” OR “online portal” OR e-platform OR “online platform” OR “web based platform” OR “digital platform” OR wearable OR biofeedback OR comput* OR gamification OR game* OR gaming OR videogame OR “artificial intelligence” OR AI OR “augmented reality” OR AR OR e-health OR web OR cyber OR multimedia OR remote* OR app OR application OR “mobile technology” OR “mobile device” OR “mobile platform” OR smartphone OR phone OR “handheld device” OR “handheld application” OR interactive OR “digital device”  AND  “emotion* regulation” OR “emotion* management” OR “affect regulation” OR “self control” OR “self regulation” OR “emotion* control” OR “affective self control” OR social cognition OR perspective taking OR metacognition OR mentalis* OR mentaliz* OR TOM OR theory of mind |
| IEEE Xplore | :(("emotion regulation" OR "emotional regulation" OR "emotional management" OR "emotion management" OR "affect regulation" OR "self control" OR "self regulation" OR "emotion control" OR "emotional control" OR "affective self control" OR "social cognition" OR "perspective taking" OR “metacognition” OR “mentalis*” OR “mentaliz*” OR “TOM” OR "theory of mind") AND (adolescent OR adolescence OR child OR children OR teenage OR teenager OR teenagers OR youth OR youths) AND (intervention OR interventions OR strategy OR strategies OR train OR training OR trained OR teach OR placebo OR placebos OR programme OR program OR programs OR programmes OR prevent OR preventative OR therapy OR therapeutic OR treatment OR treat OR treated) AND (internet OR virtual OR online OR neurofeedback OR portal OR "e portal" OR "digital portal" OR "web based portal" OR "online portal" OR "e platform" OR "online platform" OR "digital platform" OR "web based platform" OR wearable OR biofeedback OR technology OR computer* OR gamification OR game OR videogame OR "artificial intelligence" OR "augmented reality" OR "e health" OR digit* OR web OR cyber OR multimedia OR remote OR remotely OR application OR app OR "mobile technology" OR "mobile device" OR "mobile platform" OR smartphone OR phone OR "handheld device" OR "handheld application" OR interactive OR "digital device*")) |
