# Supplementary material for: Digital Interventions for Emotion Regulation in Children and Early Adolescents: Systematic Review and Meta-analysis
Source: JMIR Serious Games. 2022 Aug 19;10(3):e31456. doi: 10.2196/31456 (PMC9440412; doi:10.2196/31456)
Supplement: Multimedia Appendix 3 [file games_v10i3e31456_app3.docx]

**Multimedia Appendix 3. Risk-of-bias tool domains and definitions.**

| **RoB domain** | **RoB source** | **Low risk of bias definition for review** | **Yes, no, unclear or N/A with example of associated extraction table statement** |
| --- | --- | --- | --- |
| Selection bias | 1. Random sequence generation | Randomisation was employed to allocate participants to intervention ***and*** the random sequence generation method was clearly explained (where, using what method, with what software). | Yes: Randomisation was employed to allocate participants to intervention and the randomisation lists were obtained using x procedure (explain this clearly), at x location.  No: Randomisation was not employed to allocate participants to intervention *or* randomisation was used BUT evidence for generation of a randomised sequence was not provided.  Unclear: The randomisation lists were created at x but further details were not provided.  N/A: The study design employed was not relevant to random sequence generation selection bias, e.g. single case study or feasibility study. |
|  | 1. Allocation concealment | Randomisation was employed to allocate participants to intervention ***and*** the method used to conceal the allocation sequence from the researcher was explained clearly. | Yes: Randomisation was employed to allocate participants to intervention and x method was used to conceal the allocation sequence (explain this clearly); this was implemented by x.  No: Randomisation was not employed to allocate participants to intervention *or* the allocation to intervention was not concealed before intervention assignment.  Unclear: The allocation sequence was concealed but further details were not provided.  N/A: The study design employed was not relevant to allocation concealment selection bias, e.g. single group repeated measures study. |
|  | 1. Population representation | It was clear from the recruitment method that participants recruited for the study were representative of the population from which they were drawn. | Yes: Participants recruited for the study were representative of the population from which they were drawn, (e.g. five randomly selected children’s homes from a whole population of children’s homes in Scotland were included in the study or stratified sampling or systematic sampling).  No: Participants recruited for the study were not representative of the population from which they were drawn (e.g. opportunistic/convenience sampling at a youth wellbeing drop-in group in x city suburb used to recruit a looked-after children population or self-selecting sample).  Unclear: Recruitment method is unclear, *or* participants are fairly typical of the average in the population from which they were drawn (e.g. looked-after children population).  N/A: The study design employed was not relevant to population representation selection bias, e.g. RCT. |
| Performance bias | 1. Blinding of participants, raters and intervention deliverer* | Measures are used to blind participants, raters and intervention deliverer(s) from knowledge of which intervention participants received and these were explained; ***or*** (measures were used to blind participants from knowing that the authors wished to create a satisfactory intervention/assess part of an intervention **applicable to feasibility/acceptability type studies only*) ***and*** information relating to whether the intended blinding was effective was provided. | Yes: Participants, raters and intervention deliverer(s) taking part in the feasibility study were advised they would be taking part in research on x but full aim of the study (i.e. to find out if a part of an intervention was satisfactory) was not divulged (clearly explain the relevance of the type of study in relation to the definition). The efficacy data showed x.  No: Participants and/or raters and/or intervention deliverer(s) were not blinded from knowledge of which intervention participants received in the RCT (clearly explain the relevance of the type of study in relation to the definition).  Unclear: The blinding measures were unclear. |
|  | 1. Acquiescence | In studies examining new interventions or components of interventions, methods taken to ensure that outcome assessments objectively seek opinions rather than suggesting that that one answer is desirable are described clearly ***and*** Information pertaining to whether these measures were effective is also provided. | Yes: X procedure was used in the case study to ensure that participants did not feel pressured into giving certain responses (explain this clearly). The efficacy data showed x.  No: A procedure was not put in place to ensure that participants did not feel pressured into giving certain responses in the acceptability single group study.  Unclear: It is not clear how effective the measures used to ensure that participants did not feel pressured into giving certain responses were as efficacy data was not provided.  N/A: The study design employed was not relevant to acquiescence performance bias, e.g. RCT. |
| Detection bias | 1. Blinding/objectivity of outcome measures* | The person(s) interpreting the data was not aware of the hypotheses and aims; information was not accessible to them to allow them to be able to foresee the outcome (e.g. group affiliation data) ***and*** information concerning whether this was effective was provided *or* the outcomes were objective e.g. time taken to maintain an oscillatory frequency above a specified threshold. | Yes: The methods used to blind the person(s) interpreting the data from knowledge of the study hypotheses, aims and information pertaining to likely outcome of participants result were x (clearly explain this). The efficacy data showed x.  No: The person(s) interpreting the data were not blinded from knowledge of the hypotheses and aims and which intervention participants received.  Unclear: The blinding (and/or) objectivity of all outcome measures were unclear. |
| Attrition bias | 1. Incomplete outcome data* | Data was provided for all outcome variables. For each outcome measure, attrition (<15% total across all available data) ***and*** exclusions from analysis data was provided with reasons or ITT was used (including the numbers in each intervention group (compared with total participants), ***and*** any re-inclusions in analyses for the review; ***or*** the study design employed resulted in complete outcome data e.g. single case study. | Yes: Data was provided for all outcome variables *and* <15% attrition (give specific %). This was due to x. n = x lost in x group, n = x lost in x group; total participants = x.  No: Data was not provided for all outcome variables *and/or* >15% attrition (give specific %). No information regarding exclusions provided and no information provided related to reasons, or breakdown for each intervention group.  Unclear: The attrition data was not provided or was unclear.  N/A: The study design employed was not relevant to attrition bias, e.g. a study examining a component part of an intervention. |
| Reporting bias | 1. Selective reporting* | Selective outcome reporting was documented ***and*** the findings were presented. | Yes: There are no discrepancies between measures used and outcome data; *or* any discrepancies between the measures and outcome data are clearly justified (document justification).  No: There are discrepancies between measures used and outcome data *and* justification information in relation to selective outcome reporting was not provided. |
|  | 1. Baseline outcome measurements similar* | Performance or clinical outcomes were measured before the intervention in non-randomised trials, ***and*** there were no significant differences across groups, ***or*** there were differences across groups in randomised trials but this was taken into account in the analysis (e.g. ANCOVA). | Yes: Performance in x and x were measured at baseline in the non-randomised trial and there were no significant differences between groups; *or* performance in x and x were measured at baseline in the randomised trial and significant differences observed between groups was taken into account in the statistical analysis (report statistical method used).  No: Important differences were found in baseline performance scores in the non-randomised trial; *or* there were differences between groups in the randomised trial and this was not taken into account in the analysis.  Unclear: Baseline performance was measured, however data was not provided.  N/A: The study design employed was not relevant to baseline outcome measurements similar reporting bias, e.g. single group repeated measures design. |
|  | 1. Validation and reliability of outcome measures* | All outcome measures were validated and/or reliable, as evidenced in the text or through further investigation into the outcome measure(s). | Yes: All outcomes measures were validated and/or reliable (report validity and reliability data for each outcome measure); for example: acceptable factor analysis loading values for validity and/or Cronbach’s α values for reliability.  No: Some, but not all outcome measures were validated and/or reliable (report available validity and reliability data for each outcome measure); for example: acceptable factor analysis loading values for validity and/or Cronbach’s α values for reliability. |
|  | 1. Full-scale study criteria transparency | The criteria used in feasibility, pilot or single case studies to determine whether to conduct a full-scale study were provided (as well as results of all outcome measures) ***and*** the outcome and implications of this were clearly documented. | Yes: The criteria that was employed to determine whether to take the current study to a full-scale study were: x, x and x. The outcome of this was: x, the implication of this was: x.  No: Criteria used to determine whether to take the current study to a full-scale study was not provided.  Unclear: The criteria that was used to determine whether to take the current study to a full-scale study were: x, x and x, however the outcome of this was not provided *or* were unclear.  N/A: The study design employed was not relevant to future research criteria transparency, e.g. RCT. |
| Other bias(s) | 1. e.g. **Seasonality**, time of measurement, maturation, mortality, intervention setting differences, extreme high or low score at baseline (regression to mean effects), measurement differences (different outcome measure for different type of intervention). | There was no evidence of other sources of bias (i.e. caused by an extraneous variable) not accounted for by clearly described, specific methods, not previously covered in the other 5 domains. | Yes: There was no evidence of other sources of bias.  No: A spurious effect may have been caused, e.g. by seasonal differences; the baseline measures were completed in January and the post intervention measures were completed in August.  Unclear: There were potential spurious effects of x and x, however these were unclear. |
|  | 1. Competing interest and source of support | The author clearly stated that there were no competing interests **and** documented any sources of support (i.e. funding). | Yes: There were no competing interests and the source(s) of support are documented.  Partial: Only the competing interest information *or* only the source of support was documented by the author.  No: The competing interest and source of support was not documented by the author. |

*=To assess each main outcome or class of outcomes.

**Scoring**

- Yes = 2 points
- No = 0 points
- Unclear/partial = 1 point
- N/A = 2 points

Max quality score = 26

>80% (>21) = high quality

>60% (>16) = moderate quality

<59% (<15) = low quality
