# Supplementary material for: Digital Interventions for Emotion Regulation in Children and Early Adolescents: Systematic Review and Meta-analysis
Source: JMIR Serious Games. 2022 Aug 19;10(3):e31456. doi: 10.2196/31456 (PMC9440412; doi:10.2196/31456)
Supplement: Multimedia Appendix 4 [file games_v10i3e31456_app4.docx]

Online Supplementary Material Four.

Emotion regulation digital intervention characteristic matrix.

*Note.* Studies presented based on intervention type:
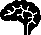
 = biofeedback;
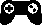
 = digital game;
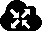
 = virtual and augmented reality;
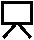
 = programme and multimedia. Comparison information for studies included in meta-analytic component only. TAU= treatment as usual control; WL=waitlist control. Where a study reported on multiple samples, characteristics for each sample are presented separately. EEG α/θ frequency associated with relaxation; EEG β frequency associated with focus; EEG high β frequency associated with anxiety; HR, HRV and cardiac coherence associated with emotional arousal; SCP=slow cortical potential; RPG=role player game; ER=emotion regulation; PTSD=post-traumatic stress disorder; ADHD=attention deficit hyperactivity disorder; ASD=autism spectrum disorder; VR=virtual reality; AR=augmented reality. ABM=attention bias modification; PMR=progressive muscle relaxation; CBT=cognitive behavioural therapy; ACT=anger control therapy; REBT=rational emotive behaviour therapy; MCT=mindfulness cognitive therapy; BCI=brain computer interaction; HR=heart rate; GSR=galvanic skin response. NF=neurofeedback; BF=biofeedback. **-**=not reported.

| Study & (reference number) | Digital intervention | Additional component | *M* age | Sample | *N* (Intervention/control/additional control)) | Theory & intervention description | Setting | Length/ session *N* |
| --- | --- | --- | --- | --- | --- | --- | --- | --- |
| 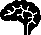 Cohen2016 (56) | fMRI NF |  | 11.6 | Healthy | 19 | Increase or decrease anterior insular fMRI amplitude/thermometer dial via thinking happy thoughts or relaxing. | University research centre | One day/ four 3-min sessions |
| 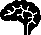 Torrado 2017 (79) | LG Watch Urbane HR BF**^c^** | Caregiver support & computer | 10 | ASD (low function) | 2 | Reduce HR below threshold during emotional outburst via alert and caregiver-designed personalised ER prompt. | School class | Nine days/ nine 4-hr sessions |
| 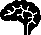  **^a^**Lackner 2016 (68) | EEG NF^d^ |  | - | Anorexia Nervosa | 20 (10/ 10) | *Theory of operant conditioning of brain oscillations.* Maintain EEG α above threshold to raise ball over line via self-chosen cognitive strategies. | Hospital therapy room | Five weeks/ ten 20-min sessions |
| 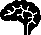  **^b^**Goodman 2018 (63) | Thought Technology HR BF & EEG NF**^c^** | Researcher strategy modelling | 12.4 | ASD (mixed function) | 15 | *Operant conditioning of brain oscillations & BCI.* Maintain EEG α-mu power above and breathing rate below thresholds to play DVD via diaphragmatic breathing. | Unspecified clinical setting | Six weeks/ twelve 80-min max. sessions |
| 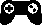  **^b^**Heinrich 2020 (64) | SAM^e^ | Real-life strategy practice | 9.8 | ADHD | 48 (30/ 18) | *Neurobehavioural approach.* EEG NF. Maintain relative θ/β or SCP to win games via self-chosen cognitive strategies. | Unspecified clinical setting | Four weeks/ 36 50-min sessions |
| 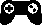  **^ab^**Rogel 2020 (71) | EEGer4 & Zukor Interactive^d^ |  | 9.6 | PTSD | 32 (16/ 16) | *BCI.* EEG NF. Maintain posterior α to win audio/visual rewards via passive game interaction. | Therapy centre | Twelve weeks/24 18-min max. sessions |
| 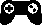 Filella 2016 (61) | Happy 8–12**^c^** |  | 10.5 | Universal | 574 (351/ 223) | *Modal model of emotion.* RPG. Resolve conflicts by choosing correct assertive response from list. | **-** | 30 1-hr sessions |
| 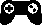 Filella 2018 (62) | Happy 12–16**^c^** |  | 12.6 | Universal | 903 (472/ 431) | *Modal model of emotion.* RPG. Resolve conflicts by choosing correct assertive response from list. | School tutor sessions | 30 1-hr sessions |
| 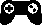 David 2018 (57) | ReThink mini-game | Therapist REBT description | 13 | Healthy | 25 | *REBT.* 2D. Learn about and practice differentiating between emotions to win keys and territories. | Unspecified group setting | One day/ one 30-min session |
| 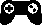  **^a^**David 2019 (58) | ReThink |  | 12.9 | Healthy | 142 (48/ 48/ 46) | *REBT.* 2D. Learn about and practice REBT-based strategies to win keys and territories. | School class | Four weeks/ seven 50-min sessions |
| 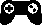  **^a^**David 2020 (59) | ReThink |  | 12.9 | Healthy | 134 (47/ 42/ 45) | *REBT.* 2D. Learn about and practice REBT-based strategies to win keys and territories. | School class | Four weeks/ seven 50-min sessions |
| 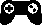 Rodriguez 2015 (70) | Game-Teen System | Bluetooth therapist monitoring | **-** | Healthy | 52 | *Theory of embodied cognition.* VR-enabled 3D frustration induction. Breathe with undulating feather & identify incorrect numbers in sequence. | **-** | One day/ one 45-min session |
| 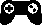 Vara 2016a (80) | Game-Teen System |  | 13 | Healthy | 61 | *Theory of embodied cognition.* VR-enabled 3D frustration induction. Breathe with undulating feather. | School | One day/ one 20-min session |
| 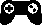 Vara 2016b (81) | Game-Teen System |  | 13.6 | Healthy | 63 | *Theory of embodied cognition.* VR-enabled 3D joy induction. Breathe with undulating feather. | **-** | One day/ one 20-min session |
| 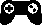  **^b^**Antle 2018 (49) | Mind-Full**^c^** | Bluetooth therapist monitoring/ support | - | Living in poverty | 21 (9/ 12) | *MCT.* EEG NF 2D. Maintain α/θ or β above threshold to control pinwheel and paraglider or build stone stack via body relaxation/deep breathing or sustained attention. | School counsellor sessions | Six weeks/ 24 15-min sessions |
| 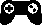 Kahn 2013 (67) | RAGE-Control^d^ | ACT & therapist support | - | Elevated anger/ aggression | 37 (18/ 19) | *CBT & ACT.* BF 2D. Maintain HR below threshold to navigate spaceship and shoot aliens via deep breathing. | Inpatient psychiatric unit | Five days/ five 30-min sessions |
| 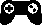 Lutz 2014 (69) | HeartMath HRV^d^ | Therapist support | - | Emotionally disturbed offenders | **-** | BF 2D. Maintain high cardiac coherence to make rainbow drop coins into vessel via positive focus and rhythmic breathing. | Psychiatric unit | **-** |
| 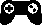  **^a^**Schuurmans 2018 (75) | Dojo^d^ |  | 13.9 | Anxiety with/ without ID | 37 (18/ 19) | *CBT.* Immersive BF 3D fear, frustration & anger induction with character-led tutorials. Maintain HR below threshold to win mini-games via deep breathing, PMR, positive thinking, guided imagery. | Residential home | Four weeks/ eight 30-min sessions |
| 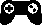  **^a^**Scholten 2016 (73) | Dojo^e^ |  | 13.3 | Elevated anxiety | 138 (70/ 68) | *CBT.* Immersive BF 3D fear, frustration & anger induction with character-led tutorials. Maintain HR below threshold to win mini-games via deep breathing, PMR, positive thinking, guided imagery. | School & home | Three weeks/six 1-hr sessions |
| 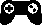  **^a^**Schoneveld 2016 (46) | Mindlight^e^ |  | 9.9 | Elevated anxiety | 136 (69/ 67) | *CBT.* Immersive, EEG-NF 3D anxiety induction with guidance. Maintain high β below and α above threshold to navigate scenarios in old mansion via deep breathing, self-talk and ABM. | School | Three weeks/five 1-hr sessions |
| 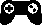  **^a^**Schoneveld 2018 (47) | Mindlight^e^ |  | 9.9 | Elevated anxiety | 174 (86/ 88) | *CBT.* Immersive, EEG-NF 3D anxiety induction with guidance. Maintain high β below and α above threshold to navigate scenarios in old mansion via deep breathing, self-talk and ABM. | School | Six weeks/ six 1-hr sessions |
| 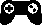  **^a^**Schoneveld 2020 (74) | Mindlight^e^ |  | 9.9 | Elevated anxiety | 174 (86/ 88) | *CBT.* Immersive, EEG-NF 3D anxiety induction with guidance. Maintain high β below and α above threshold to navigate scenarios in old mansion via deep breathing, self-talk and ABM. | School | Six weeks/ six 1-hr sessions |
| 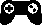 **^a^**Wijnhoven 2020 (45) | Mindlight^d^ | Therapist psychoeducation and support | 11.1 | ASD (high function) elevated anxiety | 109 (53/ 56) | *CBT.* Immersive, EEG-NF 3D anxiety induction with guidance. Maintain high β below and α above threshold to navigate scenarios in old mansion via deep breathing, self-talk and ABM. | School individual session | Six weeks/ six 1-hr sessions |
| 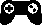 **^b^**Beaumont 2008 (50) | Secret Agent Society**^c^** | Homework, group work, parent training | 9.7 | ASD (high function) | 49 (26/ 23) | *Optimising ASD SS Programmes.* 3D RPG with character-led guidance. Decode feelings, complete social problem solving missions, choose correct defeat strategy to graduate spy school. | University research centre | Seven weeks/eight 2-hr sessions+ booster & parent sessions |
| 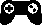 **^b^**Beaumont 2015 (51) | Secret Agent Society^d^ | Homework, classwork | 9.5 | ASD (high function) | 69 | *Optimising ASD SS Programmes.* 3D RPG with character-led guidance. Decode feelings, complete social problem solving missions, choose correct defeat strategy to graduate spy school. | *S*chool | Ten weeks/ ten 90-min sessions |
| 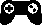 **^b^**Sofronnoff 2017 (78) | Secret Agent Society**^c^** | Delivered by parents. Homework, parent training | 9.6 | ASD (high function) | 41 | *Optimising ASD SS Programmes.* 3D RPG with character-led guidance. Decode feelings, complete social problem solving missions, choose correct defeat strategy to graduate spy school. | Home | 12-18 weeks/ten 90-min sessions |
| 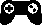 **^b^**Einfield 2017 (60) | Secret Agent Society**^c^** | Homework, classwork, parent training | 10.7 | ASD (mixed function) | 84 (26/ 58) | *Optimising ASD SS Programmes.* 3D RPG with character-led guidance. Decode feelings, complete social problem solving missions, choose correct defeat strategy to graduate spy school. | School | 10-13 weeks/ten 90-min sessions+ booster & parent sessions |
| 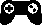 **^b^**Beaumont 2019 (52) | Secret Agent Society^e^ | Homework, group work, parent training | 9.8 | Peer difficulties/social anxiety | 27 | *Optimising ASD SS Programmes.* 3D RPG with character-led guidance. Decode feelings, complete social problem solving missions, choose correct defeat strategy to graduate spy school. | University research centre | Nine weeks/ nine 90-min sessions+ booster & parent sessions |
| 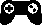 **^b^**Shum 2019 (76) | Adventures of DoReMiFa**^c^** | Classwork | 9.5 | Universal | 332 (220/ 112) | *CBT & positive psychology.* 2D modules with characters representing skills/deficits. Read stories and win challenges to find hidden book. | School classes | 4–6 months/ eleven 20-min sessions |
| 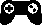 Carlier 2020 (53) | New Horizon**^c^** | Tracking & supportive parent app | 8 | ASD (high function) | 3 | *NICE modified CBT for children with ASD.* 2D. Collect stardust and snacks via guided imagery, visualisation and deep breathing. Supplemented with non-therapeutic mini-games | Home | Two weeks/no set sessions |
| 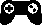 Amon 2008 (48) | Journey to Wild Divine**^d^** | Researcher motivation & guidance | 9.5 | ADHD | 24 | *BF therapy.* BF, GSR commercial with character-led tutorials. Maintain HR below threshold to navigate island via breathing techniques. | University research centre | Twelve weeks/12, 18 or 24 45-min sessions |
|  |  |  | 8.8 | Healthy control | 12 | *BF therapy.* BF, GSR commercial with character-led tutorials. Maintain HR below threshold to navigate island via breathing techniques. | University research centre | Twelve weeks/12, 18 or 24 45-min sessions |
| 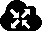 Wrzesien 2015 (82) | 3DMeNow Pro™ |  | 13 | Healthy | **-** | *Modelling therapy*. Immersive VR. Watch self-representing avatar become frustrated with computer and use focused breathing strategy. | School | One day/ one 30-min session |
| 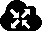 **^b^**Ruiz-Ariza 2018 (72) | Pokémon GO |  | 13.3 | Healthy | 190 (87/ 103) | Commercial AR outdoor quest. Catch Pokémon and us them to fight other players. | Outside (e.g. parks) | Eight weeks/ no set sessions |
| 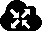 **^b^**Yuan 2018 (83) | CAVE**^c^** | Trainer guidance/ debrief | 8.9 | ASD (high function) | 72 (36/ 36) | *Cognitive theory of multimedia learning & group therapy.* Immersive VR. Navigate group control, relaxation and social scenarios via adaptive emotional and behavioural responses. | University research centre | Weeks unspecified/twelve 1-hr sessions |
| 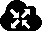 **^b^**Ip 2018 (66) | Half-CAVE**^c^** | Observation, trainer guidance/ debrief & worksheets | 9 | ASD (high function) | 72 (36/ 36) | *Cognitive theory of multimedia learning & group therapy.* Immersive VR. Navigate control, relaxation and social scenarios via adaptive emotional and behavioural responses. | **-** | Fourteen weeks/28 1-hr sessions |
| 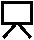 **^b^**Carroll 2016 (54) | KOOL-Kids**^c^** | Classwork, homework & finale celebration | 8.9 | Suspended/at risk of school suspension | 49 | *CBT model of aggressive and antisocial behaviour.* Multimedia modular programme. Learn about emotions, identity, social skills and strategies with animated stories. | School | Twelve weeks/ thirteen 1-hr sessions |
| 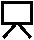 **^b^**Houghton 2017 (65) | KOOL-Kids**^c^** | Classwork, homework & finale celebration | 10 | Excluded/ suspended from school | 13 | *CBT model of aggressive and antisocial behaviour.* Multimedia modular programme. Learn about emotions, identity, social skills and strategies with animated stories. | School | Twelve weeks/ thirteen 1-hr sessions |
| 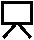 **^b^**Carroll 2020 (55) | KOOL-Kids**^c^** | Classwork, homework & finale celebration | 9.7 | Universal | 854 (562/ 292) | *CBT model of aggressive and antisocial behaviour.* Multimedia modular programme. Learn about emotions, identity, social skills and strategies with animated stories. | School | Twelve weeks/ thirteen 1-hr sessions |
| 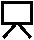 **^ab^**Smith 2018 (77) | Emotion Theorie^d^ |  | **-** | Universal | 1645 (831/ 814) | *Implicit theories of emotion.* Online programme. Learn about emotions, beliefs about the nature/malleability of them and ER strategies, with scenarios and questions. | School | 2-4 weeks/ two 45-min sessions |

^a^=included in meta-analysis

**^b^**=intervention also trained other skill(s)

^c^=continuance of existing treatment unclear

^d^=continuance of existing treatment permitted

^e^=continuance of existing treatment not permitted
