# Supplementary material for: Digital Interventions for Emotion Regulation in Children and Early Adolescents: Systematic Review and Meta-analysis
Source: JMIR Serious Games. 2022 Aug 19;10(3):e31456. doi: 10.2196/31456 (PMC9440412; doi:10.2196/31456)
Supplement: Multimedia Appendix 5 [file games_v10i3e31456_app5.docx]

**Multimedia Appendix 5. Intervention characteristic summary.**

**Biofeedback**

*LG Watch Urbane Heart rate biofeedback* (n = 1), with an unspecified theoretical or therapeutic basis, was used to train ER in youth with low functioning ASD. It was engaged with in school on a smartwatch over 9 days for 4 hours a day. Participants reduced their heart rate during emotional outbursts by using caregiver led personalised calming strategies (e.g. counting, asking for hugs) with assets such as music and animations. Caregiver-led behavioural intervention and computer for strategy engagement was also provided.

*Thought Technology Heart rate biofeedback and EEG neurofeedback* (n = 1), informed by the theory of operant conditioning of brain oscillations and BCI was used to train ER, social cognition, social skills and address other specific ASD symptomatology in youth with mixed functioning ASD. It was engaged with in a clinical setting on a computer over 6 weeks, with two 80-minute (maximum length) sessions per week. Participants made a DVD play by maintaining alpha-mu power above, and breathing rate below, a specific threshold via diaphragmatic breathing. Researcher-led modelling of diaphragmatic breathing was also provided.

*EEG neurofeedback* (n = 1), informed by the theory of operant conditioning of brain oscillations was used to train ER and address low mood and specific AN symptomatology in youth with AN. It was engaged with in a clinical setting on a computer over 5 weeks, with two 20-minute sessions per week. Participants raised a ball over a line and changed its colour by maintaining alpha power above a specific threshold via undefined self-chosen cognitive strategies. No additional support or non-digital delivery was provided.

*fMRI neurofeedback* (n = 1), with an unspecified theoretical or therapeutic basis, was used to train ER and ER network connectivity in healthy youth. It was engaged with in a research institute on a computer over four 3-minute sessions in one day. Each session consisted of 5 20-second down-regulation blocks and 4 20-second up-regulation blocks. Participants increased and decreased the dial on a thermometer by increasing and decreasing the amplitude of the fMRI signal in the right anterior insular by thinking happy thoughts, and relaxing, respectively. No additional support or non-digital delivery was provided.

**Digital Games**

Twenty-seven studies assessed 15 different digital game interventions for ER. *Secret Agent Society* (n = 5), an intervention package including an immersive, ASD social skills programme-informed, 3D role play video game trained ER, social skills and social cognition in youth with ASD or peer relationship difficulties/social anxiety. It was engaged with in a research institute, special ASD class, at home or school on a computer over 7 – 13 weeks, with one 2-hour session a week. With guidance from a game character, participants learnt about and practiced detecting emotions in themselves and others; emotion intensity classification; detecting unhelpful cognitions; cognitive reframing; coping with emotionally difficult everyday scenarios; perspective-taking; conversation skills; social consequences, as a junior detective in a spy school. However, game mechanics were undefined. Class sessions, parent training and homework with digital journal notes were also included.

*Mindlight* (n = 4), an immersive, CBT-informed, 3D video game with EEG neurofeedback and anxiety induction trained ER for anxiety prevention in youth with and without ASD and elevated anxiety. It was played in school and after school on an Xbox 360 or computer over three weeks, with two sessions per week, or over six weeks, with one session per week. With in-game instruction, participants learnt about and practiced deep breathing, self-talk and attention bias modification in various anxiety-inducing scenarios in an old mansion. The player’s ability to control their relative beta and alpha frequency power via a 1-channel EEG dictated their ability to win the game. Therapist anxiety psychoeducation and support was only provided in one study that targeted youth with ASD.

*Game-Teen System* (n = 3), a virtual reality enabled, theory of embodied cognition informed, 3D video game with emotion induction trained ER in healthy youth. It was played in school or unspecified location on a computer, smartphone or RGB-camera over one 20-minute or 45-minute session. In one study, participants engaged in a joy induction challenge where they pricked balloons with ammunition whilst receiving encouraging messages. Next, they played a deep breathing mini-game in which they breathed in and out in time with an undulating feather. In two studies, participants first engaged in a frustration induction challenge where they hit a mole whilst receiving antagonising messages. In one of these studies participants played the deep breathing mini-game only and in the other, participants played a second mini-game in which focused attention was used to identify incorrect numbers in a sequence. This study also reported the provision of Bluetooth enabled therapist monitoring.

*ReThink* (n = 2) and *ReThink Feeling Better* *mini game* (n = 1), 2D, REBT-informed video game trained ER for emotional resilience in healthy youth. ReThink was played in school on an Apple iPad over 4 weeks, with seven sessions. The Feeling Better mini-game was played in an unspecified group setting on a computer over one 30-minute session. In the Feeling Better mini-game, participants learned about and practiced identifying and differentiating between basic/complex/functional/dysfunctional emotions to win earth territory keys. However, game mechanics were undefined. In the full ReThink game, participants engaged in the Feeling Better mini-game, as well as playing other mini-games based on understanding cognitive processes and their relationship with emotions/behaviour; cognitive reframing; problem-solving; relaxation, to win earth territory keys. Again, game mechanics were undefined. No additional support or non-digital delivery was provided in the full ReThink game, but therapist description of REBT was provided in the Feeling Better mini-game.

*Dojo* (n = 2), an immersive, CBT-informed, 3D video game with heart rate biofeedback and emotion induction trained ER for anxiety reduction or prevention and externalising problems in youth with anxiety with/without ID, or elevated anxiety. It was played in school and after school, or at a residential home, on a PC or laptop over 3–4 weeks, with two sessions per week. Participants learnt about skills in game character led tutorials. In an anger mini-game, participants learnt about and used positive self-talk and guided imagery in a hand-slapping contest whilst negative sentences popped up. In a frustration mini-game, participants learnt about and used muscle relaxation to maneuver a ball through a maze. In a fear mini-game, participants learnt about and practiced deep breathing to collect bones whilst evading a ghost. The player’s ability to control their heart rate dictated their ability to win the game. No additional support or non-digital delivery was provided.

*Happy 8–12* (n = 1) and *Happy 12–16* (n = 1), a Modal Model of Emotion informed role-play video game trained ER for conflict resolution in universal youth. Both Happy 8–12 and Happy 12–16 were played in school on an unspecified device for 30-hours over an unspecified period. Participants resolved 25 school and family based conflicts by choosing a correct assertive response from a list. No additional support or non-digital delivery was provided.

*The Adventures of DoReMiFa* (n = 1), a CBT and positive psychology informed modular video game trained ER, mental health knowledge, social cognition, social skills and self-esteem in universal youth. It was played in school on an unspecified device in 11 20 minutes lessons, over 4–6 months. Participants helped monsters that represented socio-emotional skills and deficits to find a mental health book in a school by winning undefined mini-games, and reading stories and dialogues dedicated to different skills. Eight classroom lessons were also provided.

*RAGE-Control* (n = 1), an anger control therapy and CBT informed 2D demanding task-based video game with heart rate biofeedback trained ER for anger and aggression in an elevated anger and aggression sample. It was played in a clinical setting on an unspecified device over five daily 30-minute sessions. Participants navigated a space ship and shot aliens by using deep breathing to maintain their heart rate within a specific threshold. Anger control therapy and therapist support was also provided.

*Mind-Full* (n = 1), a mindfulness cognitive therapy informed simple challenge video game with neurofeedback trained ER and attention in youth living in poverty. It was played in school on a computer in 24 15-minute sessions over 6-weeks. Participants played two relaxation mini-games in which they controlled a pinwheel or paraglider by relaxing their body and using breathing techniques. Participants played one attention mini-game in which they built a stone stack by maintaining sustained attention on moving stones. The player’s ability to control their alpha and theta, or beta frequency power via a 1-channel EEG dictated their ability to win the game. Networked therapist monitoring and support was also provided.

*SAM* (n = 1), neurobehavioural approach informed unspecified video games with neurofeedback trained ER and self-regulation for dysregulation in youth with ADHD. It was played in a clinical setting on a computer in 36 50-minute sessions within two 18-session blocks, over 4 weeks (with a 2–3 week break between session blocks). Participants played games and won points by maintaining a relaxed state through using undefined self-chosen mental strategies. The player’s ability to control their relative beta and theta frequency power via an unspecified number of EEG channels dictated their ability to win games. Real life strategy practice and documentation was also included.

*EEGer4* and *Zukor Interactive* (n = 1), BCI informed unspecified commercial video games with neurofeedback trained ER, executive function and addressed externalising, low mood and PTSD specific symptomatology in youth with PTSD. It was played in a clinical setting on a computer, twice a week in 24 18-minute (maximum) sessions over 12 weeks. Participants passively watched video games that reflected their EEG activity via an unspecified number of EEG channels. They won audio and visual rewards when the EEG was in their personalised posterior dominant rhythm (PDR) 3-Hz band. No additional support or non-digital delivery was provided.

*New Horizon* (n = 1), a modified CBT for children with ASD informed 2D exploration and puzzle video game trained ER for anxiety in youth with high functioning ASD. It was played daily at home on a smartphone for 2 weeks. Participants played two relaxation mini-games, one focused on guided imagery and visualisation – participants popped bubbles of specific colours within a time limit to collect stardust. One focused on relaxation – participants inhaled and exhaled at the correct time to win snacks for a space whale. Participants also played two non-therapeutic mini-games in which they recreated a sequence of stars, and, explored a new territory whilst avoiding hazards. A tracking and supportive application for parents called Space Control, and parental encouragement, were also provided.

*HeartMath HRV* (n = 1), a simple challenge video game with biofeedback with an unspecified theoretical or therapeutic basis trained ER in a young offender sample with unspecified emotional disturbance. It was played in a clinical setting on a computer over an unspecified period. Participants made a rainbow drop coins into a vessel by using positive focus and rhythmic breathing to maintain high cardiac coherence. The participant’s ability to maintain high cardiac coherence dictated their ability to win the game. Clinician support was also provided.

*Journey to Wild Divine* (n = 1), a biofeedback therapy informed commercial video game with biofeedback and galvanic skin response (GSR) trained ER for emotional symptoms and addressed specific ADHD symptomatology in healthy youth and those with ADHD. It was played for 45-minutes in a research institute on a computer once a week, twice a week or three times a week over 12 weeks. Participants learnt breathing techniques via a game character and practised them to win activities, (e.g. creating a pathway across an island). The participant’s ability to control their heart rate dictated their ability to win the game. Researcher motivation and guidance was also provided.

**Virtual Reality and Augmented Reality**

*CAVE* (n = 1) and *Half-CAVE* (n = 1), cognitive theory of multimedia learning and group therapy informed immersive virtual environments trained ER, social cognition and social skills in youth with high functioning ASD. CAVE was engaged with in a research institute in twelve 1-hour sessions over an unspecified period. In groups, participants navigated control/relaxation and social scenarios by practising adaptive emotional and behavioural responses. Half-CAVE was engaged with in an unspecified location twice a week in 28 sessions over 14 weeks. Participants were guided through the virtual environment individually in turn for 10 minutes whilst other participants observed. Within the CAVE study, trainer guidance and support were also provided; in addition, the Half-CAVE study provided observation/reflection worksheets.

*Pokémon GO* (n = 1), a commercial, augmented reality quest with an unspecified theoretical or therapeutic basis trained ER, executive function and social skills in healthy youth. It was engaged with outside environment (e.g. parks) on a smartphone over 8 weeks. Participants caught Pokémon characters superimposed to the outside environment and used them to battle other players. Higher levels were accessed by walking greater distances. No additional support or non-digital delivery was provided.

*3DMeNow Pro™* (n = 1), a modelling therapy informed immersive virtual environment with a self-representing avatar trained ER in healthy youth. It was engaged with in school on a computer in one 30-minute session. Participants watched a self-representing avatar become very frustrated with a computer then implement a focused breathing strategy. No additional support or non-digital delivery was provided.

**Programme and Multimedia**

*KOOL-Kids* (n = 3), a CBT model of aggressive and antisocial behaviour informed multimedia, modular school programme trained ER, social skills and social cognition and addressed externalising in universal youth and youth at risk of or suspended/excluded from school. It was engaged with at school on an unspecified device in 13 one-hour weekly sessions over 12 weeks. Participants engaged in four modules within five whole-of-class sessions, based on: K = know yourself; O = our needs/emotions; O = others’ needs/emotions; L = living well with others. Individual referred child sessions used eight sequentially linked animated stories in which character ‘Okki Octopus’ had difficulty managing emotions and experienced frequent peer/teacher conflicts. Here, participants learnt key strategies via Okki and explored *their* individual difficulties. Facilitator- and teacher-led sessions, homework and a finale celebration were also provided.

*Emotion Theories* (n = 1), an implicit theories of emotion informed online school programme trained ER and beliefs about the nature and malleability of emotions in a universal sample. It was engaged with at school on a computer in two 45-minute sessions over 2–4 weeks. Participants read/listened to information about what emotions are and how they form, the recognition of emotional experiences, and normalisation of ER difficulties in session one. In session two, participants read/listened to information about adaptive emotion theories and ER strategies. Common school-based emotionally difficult experiences (e.g. bullying) were the example scenarios. Interactive components (e.g. questions) allowed the practising of learnt material. No additional support or non-digital delivery was provided.
