# Supplementary material for: Digital Interventions for Emotion Regulation in Children and Early Adolescents: Systematic Review and Meta-analysis
Source: JMIR Serious Games. 2022 Aug 19;10(3):e31456. doi: 10.2196/31456 (PMC9440412; doi:10.2196/31456)
Supplement: Multimedia Appendix 6 [file games_v10i3e31456_app6.docx]

**Multimedia Appendix 6. Risk-of-bias result summary.**

Of the 27/39 (69%) studies that were applicable to *random sequence generation and allocation concealment* (selection bias), most (18/27, 67%) did not apply randomisation or a random component in the sequence generation, or there was not sufficient information to make a judgment. Of those that did (9/27, 33%), most were produced using an independent researcher and computer-based random number generator. However, only 3/9 (33%) also described appropriate allocation concealment. Of the 27/39 (69%) studies that were applicable to *population representation* (selection bias), most (26/27, 96%) either did not provide adequate information to make a judgment or used recruitment methods that resulted in an inadequately representative sample. Hence, the risk of selection bias was high. The risk of performance bias *(blinding of participants, raters and study personnel, and acquiescence)* was high – all studies were unable to perform blinding in relation to knowledge of which intervention participants received; or that the authors wished to create a satisfactory intervention or assess part of an intervention. Similarly, all studies were unable to ensure that the personnel interpreting and analysing data were unaware of the associated hypotheses and aims; or that all outcomes were objective. Therefore, the risk of detection bias was high. As shown in the figure S1, there was variability across studies and clusters in review authors’ judgments about *incomplete outcome data* (attrition bias). The biofeedback cluster presented the lowest risk of attrition bias, with 50% (n = 2) of studies judged low risk. The majority of studies (4/6, 67%) judged low risk within the digital games cluster in this domain targeted at-risk populations.

*
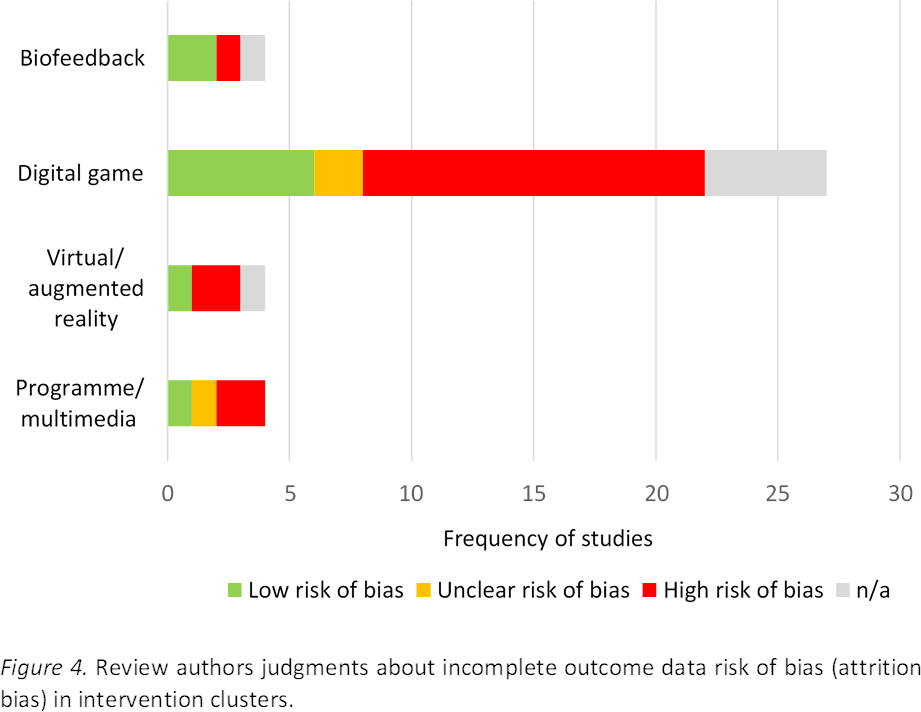
*Figure S1. Review authors judgements about incomplete outcome data risk of bias (attrition bias) in intervention clusters.

The risk of reporting bias was variable across studies. The majority of studies (36, 92%) presented no discrepancies between measures used and outcome data. However, of the 29/39 (74%) studies that were applicable to *baseline outcome measurements similar*, less than half (12, 41%) clearly demonstrated no significant differences in outcomes across groups at pre-test where the study was non-randomised; or differences across groups were taken into account where the study was randomised. The majority of these studies were in the digital game cluster. Further, the *validity and reliability* reporting bias source was deemed high risk, with less than a third 10/39 (26%) of included studies’ using only valid and reliable outcome measures in the targeted sample. In early stage studies that were applicable to *full-scale study criteria transparency* (reporting bias) (n = 10), all were high risk. That is, they did not report the criteria that would determine whether a subsequent full-scale study should be conducted, with associated outcomes. Finally, over half (23/39, 59%) of the studies appeared to demonstrate a high risk of gender bias within the other bias domain. This was evident across all intervention clusters, but largely within studies targeting at risk and diagnosed populations. The risk of bias scores were re-calculated after removing gender bias to consider whether this influenced overall quality ratings. Three studies from the digital game (n = 1), biofeedback (n = 1) and programme/multimedia (n = 1) clusters improved, moving from low quality to moderate quality overall ratings. The supplementary materials contain the Risk of Bias Matrix.
