# Supplementary material for: Digital Interventions for Emotion Regulation in Children and Early Adolescents: Systematic Review and Meta-analysis
Source: JMIR Serious Games. 2022 Aug 19;10(3):e31456. doi: 10.2196/31456 (PMC9440412; doi:10.2196/31456)
Supplement: Multimedia Appendix 7 [file games_v10i3e31456_app7.docx]

**Multimedia Appendix 7. Study and measures matrix.**

*Note.* Measures used in the meta-analysis are in bold.

| First author with year of publication | Effectiveness, efficacy, feasibility outcome measure(s) | Acceptability outcome measure(s) |
| --- | --- | --- |
| Cohen-Kadosh, 2016 | 1. Cognitive Emotion Regulation Questionnaire (Garniefski et al., 2001) (self-report) 2. fMRI Granger information flow during NFT | **-** |
| Torado, 2017 | 1. Heart rate (LG Watch Urbane™) 2. Evaluator field notes inclusive of caregiver comments |  |
| Lackner, 2016 | 1. Eyes Closed and Open Resting State EEG Band Power **2. Regulating and Controlling Own Emotions subscale of Emotional Competence Questionnaire (Rindermann 2009) (self-report)**  3. Recognising and Understanding Own Emotions subscale of Emotional Competence Questionnaire (Rindermann 2009) (self-report) 4. General Emotionality subscale of Emotional Competence Questionnaire (Rindermann 2009) (self-report) **5. Anxiety subscale of Brief Symptom Inventory shortened from Symptom Checklist-90-Revised (Franke & Derogatis, 2002; Frank 2000) (self-report)** | **-** |
| Goodman, 2018 | 1. Standard Deviation of Normal-to-Normal Wave Intervals (Thought Technology Ltd) 2. Square Root of the Mean Squared Difference of Successive Normal-to-Normal Intervals (Thought Technology Ltd) 3. Vagal tone (Thought Technology Ltd) 4. Alpha and Theta activity in resting state EEG 5. Lability/Negativity subscale of Emotion Regulation Checklist (Shields & Cicchetti, 1997) (parent-report) 6. Emotion Regulation subscale of Emotion Regulation Checklist (Shields & Cicchetti, 1997) (parent-report) 7. Children’s Anxiety Scale (Nauta et al., 2004; Spence, 1998) (parent-report) | **-** |
| Heinrich, 2020 | 1. Alpha Activity in Eyes Closed and Open Resting State EEG 2. Emotional Symptoms subscale of the Strengths and Difficulties Questionnaire (Goodman 1997) (parent report) | **-** |
| Rogel, 2020 | 1. Internalising scale of Child Behaviour Checklist (Achenbach & Rescorla, 2001) (self-report) **2. Anxiety scale of Trauma Symptom Checklist for Young Children (Briere, 2005) (self-report)** | **-** |
|  |  |  |
| Filella, 2016 | 1. Emotional Development Questionnaire (López & Pérez, 2010) (self-report) 2. State subscale of Anxiety Inventory for Children (Seisdedos, 1989; Spielberger, 1973) (self-report) | **-** |
| Filella, 2018 | 1. Emotional Development Questionnaire (López & Pérez, 2010) (self-report) 2. State subscale of Anxiety Inventory for Children (Seisdedos, 1989; Spielberger, 1973) (self-report) | **-** |
| David, 2018a | 1. In game performance (developed by study authors) | **-** |
| David, 2018b | 1. Awareness subscale of Emotion Regulation Index for Children and Adolescents (Biesecker & Easterbrooks 2001) (self-report) **2. Control subscale of Emotion Regulation Index for Children and Adolescents (Biesecker & Easterbrooks 2001) (self-report)** 3. Fear subscale of Early Adolescent Temperament Questionnaire—Revised (Ellis 2002) (self-report) **4. Emotional Symptoms subscale of the Strengths and Difficulties Questionnaire – Child Version (Goodman 1997) (self-report)** | **-** |
| David, 2020 | 1. Portable EEG (Emotiv EPOC 14-channel Headset, Emotiv Systems, Inc., San Francisco, CA) frontal alpha asymmetry **2. Concern and Anxiety subscale of Profile of Affective Distress (Opris & Macavei 2007) (self-report)** | **-** |
| Rodriguez, 2015 | 1. ECG heart rate pre-post breathing strategy 2. Thermometers scale (author unclear) (self-report) | **-** |
| Vara, 2016a | 1. Frustration Visual Analogue Scale (adapted from Stern et al., 1997) (self-report) 2. Relax Visual Analogue Scale (adapted from Stern et al., 1997) (self-report) 3. Felt Arousal Scale (adapted from Hulley et al., 2008) (self-report) | 1. Participants Report on the GT-System Scale (self-report; developed by study authors) |
| Vara, 2016b | 1. Joy Visual Analogue Scale (Stern et al., 1997) (self-report) 2. Felt Arousal Scale (Hulley et al., 2008) (self-report) | **-** |
| Antle, 2018 | 1. Portable EEG (Neurosky Mindwave Headset™) mean % time spent above 70% threshold 2. Calm instrument including open questions (developed by study authors, school counsellors and school) (school staff-report) 3. Behavioural assessment survey including open questions (developed by study authors, school counsellors and school staff) 4. Counsellor written reports and follow up emails (developed by study authors) 5. Teacher written reports (developed by study authors) 4. Observations (study authors and school staff) | 1. Counsellor written reports and follow up emails (developed by study authors) 2. Teacher written reports (developed by study authors) 3. Observations (study authors and school staff) 4. Focus groups (school staff) |
| Kahn, 2013 | 1. State Anger subscale of State and Trait Anger Expression Inventory—Child and Adolescent (Spielberger 1996) (self-report) 2. Trait Anger subscale of State and Trait Anger Expression Inventory—Child and Adolescent (Spielberger 1996) (self-report) 3. Heart rate first-last session (author unclear) | 1. Therapeutic Helpfulness Questionnaire (self-report; author unclear) |
| Lutz, 2014 | 1. Medium and high cardiac coherence during video game (emWave2™) 2. Participant verbal feedback 3. Clinician verbal feedback and observations | 1. Clinician verbal feedback and observations 2. Participant verbal feedback |
| Schuurmans, 2018 | **1. Spence Children's Anxiety Scale (Dutch version) (Spence 1998) (self-report)** 2. Spence Children's Anxiety Scale (Dutch version) (Spence 1998) (mentor report) 3. Compliance scale (developed by study authors) (self-report) | 1. User Evaluation Scale (self-report; developed by study authors) |
| Scholten, 2016 | **1. Spence Children's Anxiety Scale (Dutch version) (Spence 1998) (self-report)**  2. Game Expectations – dichotomous responses (developed by study authors) (self-report) | **-** |
| Schoneveld, 2016 | **1. Spence Children's Anxiety Scale (Dutch version) (Spence 1998) (self-report)** 2. Spence Children's Anxiety Scale (Dutch version) (Spence 1998) (parent report) 3. Game Evaluations Scale (self-report; developed by study authors) 4. Game Expectations – dichotomous response (developed by study authors) (self-report) | 1. Game Evaluations Scale (self-report; developed by study authors) |
| Schoneveld, 2018 | **1. Spence Children's Anxiety Scale (Dutch version) (Spence 1998) (self-report)** 2. Spence Children's Anxiety Scale (Dutch version) (Spence 1998) (parent report) 3. Children’s Program Ratings Scale (self-report; developed by study authors) 4. Expectations Scale (developed by study authors) (self-report) | 1. Children’s Program Ratings Scale (self-report; developed by study authors) |
| Schoneveld, 2020 | 1. Internalising subscale of Strengths and Difficulties Questionnaire – Mother Version (Goodman 1997; Stone et al. 2010) (parent report) **2. Emotion Self-Efficacy scale from Self-Efficacy Questionnaire for Children (Muris 2001) (self-report)** | **-** |
| Wijnhoven, 2020 | **1. Spence Children's Anxiety Scale (Dutch version) (Spence 1998) (self-report)** 2. Spence Children's Anxiety Scale (Dutch version) (Spence 1998) (parent report) 3. The Anxiety Disorders Interview Schedule for DSM-IV, Parent version (Siebelink & Treffers 2001) (data not provided) 4/5. Parent Expectancies for Therapy Scale (PETS; Kazdin & Holland, 1991) (self- and parent report) | **-** |
| Beaumont, 2008 | 1. James and the Maths Test (Attwood, 2004) 2. Dylan is Being Teased (Attwood, 2004) 3. Emotion Regulation and Social Skills Questionnaire (Butterworth et al., 2014) (parent report) | **-** |
| Beaumont, 2015 | 1. James and the Maths Test (Attwood, 2004) 2. Dylan is Being Teased (Attwood, 2004) 3. Emotion Regulation and Social Skills Questionnaire (Butterworth et al., 2014) (parent report) 4. Emotion Regulation and Social Skills Questionnaire (Butterworth et al., 2014) (teacher report) 5. Spence Children's Anxiety Scale (Spence 1998) (parent report) | **-** |
| Sofronoff, 2017 | 1. James and the Maths Test (Attwood, 2004) 2. Dylan is Being Teased (Attwood, 2004) 3. Emotion Regulation and Social Skills Questionnaire (Butterworth et al., 2014) (parent report) 4. Spence Children's Anxiety Scale (Dutch version) (Spence 1998) (parent report) |  |
| Einfeld, 2018 | 1. James and the Maths Test (Attwood, 2004) 2. Dylan is Being Teased (Attwood, 2004) 3. Emotion Regulation and Social Skills Questionnaire (Butterworth et al., 2014) (parent report) 4. Emotion Regulation and Social Skills Questionnaire (Butterworth et al., 2014) (teacher report) | **-** |
| Beaumont, 2019 | 1. James and the Maths Test (Attwood, 2004) 2. Dylan is Being Teased (Attwood, 2004) 3. Emotion Regulation and Social Skills Questionnaire (Butterworth et al., 2014) (parent report) 4. Spence Children's Anxiety Scale (Dutch version) (Spence 1998) (parent report) 5. Spence Children's Anxiety Scale (Dutch version) (Spence 1998) (self-report) | **-** |
| Shum, 2019 | 1. Chinese version of 9 items from Screen for Child Anxiety – Related Emotional Disorders (Birmaher 1997) (self-report) 2. Chinese version of Personal Failure subscale of Children’s Automatic Thoughts Scale-Negative or Positive, with another 10 positive items added to facilitate calculation of the state-of-mind ratios (Schniering 2002) (self-report) | **-** |
| Carlier, 2020 | 1. Spence Children’s Anxiety Scale (Scholing, 1999) (self-report) 2. Spence Children’s Anxiety Scale (Scholing, 1999) (parent report) 3. Parent Interview | 1. Parent Interview 2. In Game Mood Likert Scale (self-report; developed by study authors) |
| Amon, 2008 | 1. Strengths and Difficulties Questionnaire – composite score only (Goodman, 1997) (parent report) 2. Parent Diary 3. Game Experience Questionnaire scale (created by study authors) (parent report) | **-** |
|  |  |  |
| Wrzesien, 2015 | 1. EEG (Emotiv EPOC device™) activity across phases 2. Frustration Visual analogue scale (Adapted) (Stern et al., 1997) (self-report) 3. Relaxation Visual analogue scale (Adapted) (Stern et al., 1997) (self-report) 4. Valence subscale of Self-assessment manikin scale (Lang, 1980) (self-report) 5. Arousal subscale of Self-assessment manikin scale (Lang, 1980) (self-report) 6. Dominance subscale of Self-assessment manikin scale (Lang, 1980) (self-report) 7. Presence self-assessment manikin (Scale; Schneider et al., 2004) (self-report) 8. Identification with avatar questionnaire (adapted) (Scale; Hooi et al., 2013) (self-report) | 1. Appeal Questionnaire Scale (self-report; developed by study authors) |
| Ruiz-Ariza, 2018 | 1. Self-Control subscale of Trait and Emotional Intelligence Questionnaire, Short Form (Petrides, 2009) (self-report) 2. Emotionality subscale of Trait and Emotional Intelligence Questionnaire, Short Form (Petrides, 2009) (self-report) | 1. Satisfaction Level with Pokemon GO: Ad-Hoc Questionnaire – dichotomous response (self-report; author unclear) |
| Yuan, 2018 | 1. Affective Expressions Subtest of Psychoeducational Profile, Third Edition (Schopler et al., 2004) (parent report) 2. Ongoing Qualitative Communication Log with Parents and Teachers (Description; developed by study authors) | **-** |
| Ip, 2017 | 1. Affective Expressions Subtest of Psychoeducational Profile, Third Edition (Schopler et al., 2004) (parent report) | **-** |
|  |  |  |
| Carroll, 2017 | 1. Positive Emotions subscale of Emotional Intensity Scale for Children (Braaten & Rosén, 2000) (self-report) 2. Negative Emotions subscale of Emotional Intensity Scale for Children (Braaten & Rosén, 2000) (self-report) 3. Facilitator feedback (based on Corner et al., 2013) | 1. Acceptability Assessments Scale (self-report; developed by study authors) 2. Facilitator feedback (based on Corner et al., 2013) |
| Houghton, 2017 | 1. Positive Emotions subscale of Emotional Intensity Scale for Children (Braaten & Rosén, 2000) (self-report) 2. Negative Emotions subscale of Emotional Intensity Scale for Children (Braaten & Rosén, 2000) (self-report) 3. Facilitator feedback (based on Corner et al., 2013) | 1. Acceptability Assessments Scale (self-report; developed by study authors) 2. Facilitator feedback (based on Corner et al., 2013) 3. Acceptability Assessments Scale (facilitator report; developed by study authors) |
| Carroll, 2020 | 1. Social and Emotional Competence Questionnaire (CASEL and AIR, 2013) (teacher report) 2. Internalising subscale of Strengths and Difficulties Questionnaire – Teacher Version (Goodman 1997) (teacher report) | **-** |
| Smith, 2018 | **1. Adaptive Theories of Emotions Scale – based on Implicit Theory of Emotion Items (Tamir et al., 2007**) & Emotion Regulation Questionnaire (Gross & John, 2003) (self-report) **2. Emotional Well-Being in School Scale (developed by study authors) (self-report)** 3. Emotional Well-Being in Life Scale (developed by study authors) (self-report) | **-** |
