# Supplementary material for: Digital Interventions for Emotion Regulation in Children and Early Adolescents: Systematic Review and Meta-analysis
Source: JMIR Serious Games. 2022 Aug 19;10(3):e31456. doi: 10.2196/31456 (PMC9440412; doi:10.2196/31456)
Supplement: Multimedia Appendix 8 [file games_v10i3e31456_app8.docx]

**Multimedia Appendix 8. Meta-analysis influence analysis summary.**

The examination of influence analysis subplots indicated that two studies, (Smith et al., 2018; Schoneveld, Malmberg, Lichtwarck-Aschoff, Verheijen, Engels & Granic, 2016) presented extreme values in the emotion experience meta-analysis according to Viechtbauer and Cheung’s (2010) threshold and author visual inspection. This indicates that they may have biased the pooled effect estimate and caused some of the between-group heterogeneity. The Baujat plot indicated that the significant effect of Schoneveld et al., (2016) contributed greatly in terms of observed heterogeneity, yet its influence on the pooled effect was very small in comparison to Smith et al., 2018, which was a comparatively very large study (N = 1645) and was the only study to assess positive emotion experience. This was further corroborated within the Leave-One-Out forest plots in which the emotion experience negative pooled effect was slightly greater (*g* = -0.18) and significant (owing to non-overlapping confidence intervals) and *I*^2^ was at its lowest (*I*^2^ = 0%) when Smith et al., (2018) was removed. Of note is the non-validated and unreliable positive emotion experience measure used by Smith et al., (2018).

Considering the ER meta-analysis, examination of the influence analysis subplots indicated that Schoneveld, Wols, Lichtwarck-Aschoff, Otten and Granic (2020) presented extreme values, according to Viechtbauer and Cheung’s (2010) threshold and author visual inspection. This study may have biased the pooled effect estimate and caused some of the between-group heterogeneity. Of note is the unexpected effect observed in Schoneveld, Wols, Lichtwarck-Aschoff, Otten and Granic (2020) (in which ER was greater in the control group post-intervention). Further, this was the only non-inferiority study, and the only study that did not permit ongoing usual treatment. The extreme values observed in influence analysis subplots is supported by the Baujat plot, in which Schoneveld, Wols, Lichtwarck-Aschoff, Otten and Granic (2020) was highly influential in terms of between-study heterogeneity. Additionally, Smith et al., (2018) was highly influential in the pooled effect, yet again, this was obtained from a non-validated and non-reliable measure. This is further corroborated within the Leave-One-Out forest plots, in which the positive pooled effect was greater (g = 0.27) and significant (owing to non-overlapping confidence intervals) and *I*^2^ was at its lowest (*I*^2^ = 0%) when Schoneveld, Wols, Lichtwarck-Aschoff, Otten and Granic (2020) was removed.
