# Supplementary material for: Digital Interventions for Emotion Regulation in Children and Early Adolescents: Systematic Review and Meta-analysis
Source: JMIR Serious Games. 2022 Aug 19;10(3):e31456. doi: 10.2196/31456 (PMC9440412; doi:10.2196/31456)
Supplement: Multimedia Appendix 9 [file games_v10i3e31456_app9.docx]

**Multimedia Appendix 9. Meta-analysis trim-and-fill funnel plot.**


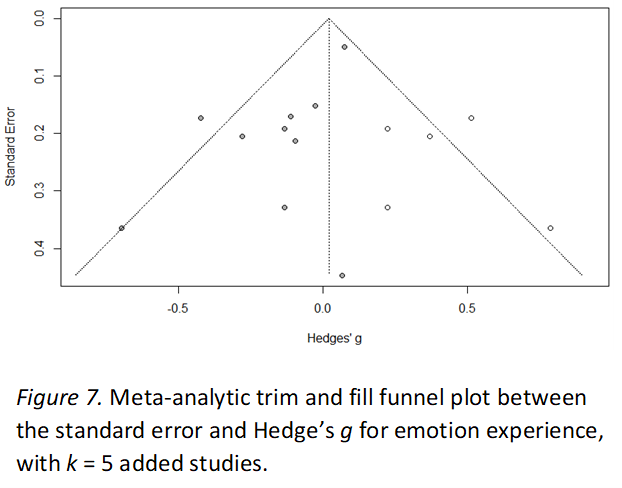
 Meta-analytic trim and fill funnel plot between the standard error and Hedge’s *g* for emotion experience, with *k* = 5 added studies.
