# Supplementary material for: Digital Interventions for Emotion Regulation in Children and Early Adolescents: Systematic Review and Meta-analysis
Source: JMIR Serious Games. 2022 Aug 19;10(3):e31456. doi: 10.2196/31456 (PMC9440412; doi:10.2196/31456)
Supplement: Multimedia Appendix 10 [file games_v10i3e31456_app10.docx]

Online Supplementary Material Ten.

Emotion regulation intervention efficacy matrix.

*Note.* This table includes efficacy outcome summaries for all studies included in the systematic review not suitable for meta-analysis.
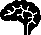
=biofeedback;
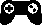
=digital game;
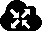
=virtual reality/augmented reality;
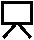
=programme/multimedia. ER=emotion regulation. EEG α/θ frequency associated with relaxation; EEG β frequency associated with focus; SCP=slow cortical potential. Green shading=low ROB; red shading=high ROB. **-**=not reported. ADHD=attention deficit hyperactivity disorder; RPG=role player game; REBT=rational emotive behaviour therapy; ACT=anger control therapy; MCT=mindfulness cognitive therapy; VR=virtual reality; AR=augmented reality. HR=heart rate; GSR=galvanic skin response. NF=neurofeedback; BF=biofeedback. See online Supplementary Material 7 for details of efficacy measures.

|  | Outcome (report type) & ROB | | |  |  |  |
| --- | --- | --- | --- | --- | --- | --- |
| Study & (reference number) | ER | Emotion experience | Physiological regulation | Outcome summary | Additional mode/*skill* | Dropout % |
| 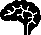 Cohen 2016 (56) | Cognitive ER scale (self) |  | fMRI ER brain network connectivity | ER: In fMRI-NF relaxation & positive thinking, only left insula up-regulation *(increased activity)* correlated significantly with cognitive ER, with a large effect (*g* = 1.26).  Physiological regulation: ER network connectivity increased significantly in upregulation only, with a medium effect (*g* = 0.52). |  | 9.5% |
| 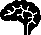 Torrado 2017 (79) |  |  | HR | Physiological regulation: In HR threshold alert & personalised ER prompt providing smartwatch, HR reduced below threshold in less than half of alerts. | Caregiver support & use of computer for strategies | **-** |
| 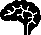 ^a^Goodman 2018 (63) | ER scale (parent) | 1. Anxiety scale (parent) 2. Lability/ negativity scale (parent) | 1. HRV (3 distinct indices) | ER: In HR-BF & HR-BF+EEG-NF threshold-based diaphragmatic breathing, ER increased significantly in HR-BF group only, with a large effect (*g* = 1.25). Emotion experience: Anxiety decreased non-significantly in both groups & lability/negativity decreased significantly in HR-BF+NF group only, with a large effect (*g* = -0.81). Physiological regulation: Significant HRV improvements in vagal tone & RMSSD in HR BF+EEG NF group only. Resting α/θ changed non-significantly in both groups. | Researcher strategy modelling/ *Social cognition* | **-** |
|  |  |  | 4. Resting EEG α/θ |  |  |  |
| ^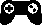 b^Heinrich 2020 (64) |  | Emotional symptom scale (parent) | Resting EEG α | Emotion experience: In SCP & θ/β EEG-NF game with self-chosen cognitive strategies, emotional symptoms decreased significantly in SCP-NF only, with a small effect (*g* = -0.41). *Change scores only.* Physiological regulation: No association between resting α & reduction in emotional symptoms. | Real-life strategy practice/*Self regulation* | **-** |
| 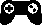 ^a^Filella 2016 (61) | Emotional competence scale (self) | State anxiety scale (self) |  | ER: In a conflict solving RPG game, emotional competence increased. Emotion experience: State anxiety decreased. |  | **-** |
| 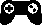 ^a^Filella 2018 (62) | Emotional competence scale (self) | State anxiety scale (self) |  | ER: In a conflict solving RPG game, emotional competence increased. *Change scores only.* Emotion experience: State anxiety decreased. *Change scores only.* |  | **-** |
| 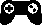 David 2018 (57) | Emotion Understanding game data |  |  | ER: In 2D REBT emotion differentiation mini-game, collection of functional emotions increased significantly after third gameplay period, with a large effect (*g* = 1.77). This diminished by the seventh period of game play. | Therapist REBT description | **-** |
| 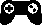 Rodriguez 2015 (70) |  | Frustration scale (self) | HR | Emotion experience: In VR 3D frustration induction & deep breathing/focused attention ER game, frustration increased significantly in high & low ER difficulty groups after induction, with a very large effect (*g* = 4.56). Frustration reduced by 72.5% across both groups after ER. Physiological regulation: HR increased significantly in both groups after induction, with a very large effect (*g* = 2.29). Non-significant decrease in HR in both groups after ER. | Bluetooth therapist monitoring | **-** |
| 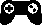 Vara 2016 (80) |  | 1. Frustration scale (self) 2. Relaxation scale (self) 3. Emotional arousal scale (self) |  | Emotion experience: In VR 3D frustration induction & deep breathing ER game using different devices, frustration increased & decreased significantly after induction & ER in phone & computer groups, respectively. But in camera group frustration decreased significantly after induction, with a large effect (*g* = -1.2) & this was maintained after ER. Arousal increased & decreased significantly after induction & ER across all groups, respectively. Relaxation decreased & increased significantly after induction & ER across all groups, respectively. |  | **-** |
| 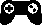 Vara 2016 (81) |  | 1. Joy scale (self) 2. Emotional arousal scale (self) |  | Emotion experience: In VR 3D joy induction & deep breathing ER game using different devices, joy increased & decreased non-significantly after induction & ER across all device groups, respectively. Arousal increased & decreased significantly after induction & ER across all groups, respectively. |  | **-** |
| ^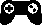 a^Antle 2018 (49) |  | Calm scale with open questions (school staff) | EEG α/θ | Emotion experience: In a MCT EEG-NF body relaxation/deep breathing ER game, feelings of calm increased significantly, with a large effect (*g* = 1.98). Reports of reduction in tearfulness, anxiety & shyness. Physiological regulation: Time spent above EEG threshold increased significantly from first 3 sessions to last 3 sessions of 2 ER games (large effect, *g* = 1.01; small effect, *g* = 0.39). | Bluetooth therapist monitoring & support/ A*ttention* | 9% |
| ^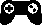 c^Kahn 2013 (67) |  | 1. State anger scale (self) 2. Trait anger scale (self) | HR | Emotion experience: In 2D HR BF deep breathing ER game, state & trait anger decreased significantly. Physiological regulation: Time spent below heart rate threshold increased significantly. | ACT & therapist support | **-** |
| ^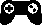 c^Lutz 2014 (69) |  | Verbal feedback (self) | Cardiac coherence | Emotion experience: In 2D HRV BF deep breathing & positive focus ER game, reports of feeling calmed. Physiological regulation: Medium or high cardiac coherence usually achieved. *Unexpected ability noted in some highly dysregulated youth.* | Therapist support | **-** |
| ^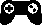 a^Beaumont 2008 (50) | 1. ER knowledge 2. ER knowledge |  |  | ER: In 3D ER, social cognition & social skill RPG with allied group-based learning sessions, anger & anxiety ER knowledge increased significantly, with large effects (*g* = 1.06;  *g* = 1.51), respectively. ER & social skills improved significantly, with a very large effect (*g* = 2.22). | Homework group work & parent training/ *Social cognition, social skills* | **-** |
|  | 3. ER & social skills scale (parent) |  |  |  |  |  |
| 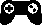 ^c^Beaumont 2015 (51) | 1. ER knowledge 2. ER knowledge | Anxiety scale (parent) |  | ER: In 3D ER, social cognition & social skill RPG with allied structured or unstructured class-based learning sessions, anxiety ER knowledge improved significantly in structured & unstructured groups & maintained at follow-up, with large (*g* = 1.5) & medium *(g* = 0.74) effects, respectively. Anger ER knowledge improved significantly in structured group only & maintained at follow-up, with a large effect (*g* = 1.47). ER & social skills improved significantly in both groups & maintained at follow-up, with medium & large effects for structured group (*g* = 0.75; *g* = 0.82) & small effects for unstructured group (*g* = 0.39; *g* = 0.45). Emotion experience: Anxiety decreased significantly in structured group only & maintained at follow-up, with a small effect (*g* = -0.36). | Homework classwork/ *Social cognition, social skills* | 1.4% |
|  | 3. ER & social skills scale (parent) 4. ER & social skills scale (teacher) |  |  |  |  |  |
| ^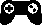 a^Sofronnoff 2017 (78) | 1. ER knowledge 2. ER knowledge | Anxiety scale (parent) |  | ER: In 3D ER, social cognition & social skill RPG with allied home-based learning sessions, only anger ER knowledge improved significantly, but by follow-up both anger & anxiety had improved significantly, with large effects (*g* = 0.92; *g* = 1.19), respectively. ER & social skills improved significantly & maintained at follow-up, with a large effect (*g* = 1.36). Emotion experience: Anxiety decreased significantly & maintained at follow-up, with a small effect (*g* = -0.48). | Delivered by parents. Homework & parent training/ *Social cognition, social skills* | 32% |
|  | 3. ER & social skills scale (parent) |  |  |  |  |  |
| 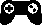 ^a^Einfield 2017 (60) | 1. ER knowledge 2. ER knowledge |  |  | ER: In 3D ER, social cognition & social skill RPG with allied class-based learning sessions, anger & anxiety ER knowledge improved significantly, with medium effects (*g* = 0.65; *g* = 0.55), respectively & maintained at follow-up. Parent-report ER & social skills improved significantly with a large effect (*g* = 1.03) & maintained at follow-up. Teacher report significant at follow-up only. *Follow-up mean/SD data unclear therefore g reported at post-intervention only.* | Homework classwork & parent training/ *Social cognition, social skills* | **-** |
|  | 3. ER & social skills scale (parent) 4. ER & social skills scale (teacher) |  |  |  |  |  |
| 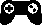 ^b^Beaumont 2019 (52) | 1. ER Knowledge 2. ER Knowledge | 1. Anxiety scale (self) 2. Anxiety scale (parent) |  | ER: In 3D ER, social cognition & social skill RPG with allied group-based learning sessions, anger & anxiety ER knowledge improved significantly, with large effects (*g* = 1.81; *g* = 2.01), respectively & maintained at follow-up. ER & social skills increased significantly & maintained at follow-up, with a large effect (*g* = 1.47). Emotion experience: Only parent-report anxiety decreased significantly & maintained at follow-up, with a large effect (*g* = -0.88). | Homework group work & parent training/ *Social cognition, social skills* | **-** |
|  | 3. ER & social skills scale (parent) |  |  |  |  |  |
| 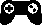 ^a^Shum 2019 (76) |  | 1. Anxiety scale (self) 2. Negative Thinking scale (self) |  | Emotion experience: In 2D modular ER, social cognition, social skill & mental health game with allied class-based learning, anxiety did not decrease & negative thinking decreased non-significantly. | Classwork/ *Social cognition, social skills, self-esteem, mental health knowledge* | 29.2% |
| ^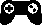 a^Carlier 2020 (53) |  | 1. Anxiety scale (self) |  | Emotion experience: In 2D guided imagery & deep breathing ER & non-therapeutic game, anxiety decreased in 2/3 youth. | Tracking & supportive parent app | 40% |
|  |  | 2. Anxiety scale (parent) |  |  |  |  |
| ^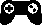 c^Amon 2008 (48) |  | Emotional, social & behavioural symptom scale (parent) |  | Emotion experience: In HR & GSR BF breathing strategy ER game (ADHD sample), symptoms decreased significantly in once-a-week group with a large effect (*g* = -1.25) & non-significantly in more than once-a-week group. | Researcher motivation & guidance | **-** |
|  |  | Emotional, social & behavioural symptom scale (parent) |  | Emotion experience: In HR & GSR BF breathing strategy ER game (healthy sample), symptoms decreased non-significantly in both groups. | Researcher motivation & guidance | **-** |
| 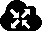 Wrzesien 2015 (82) |  | 1. Frustration scale (self) 2. Relaxation scale (self) 3. Valence scale (self) 4. Arousal scale (self) 5. Dominance scale (self) | EEG activity source | Emotion experience: In immersive VR frustration induction with self-representing (VRS group) or neutral (VRN group) avatar that modelled emotions, behaviours & ER, frustration increased & decreased non-significantly after induction & ER in VRS & VRN groups, respectively. Relaxation decreased & increased after induction & ER significantly in VRS group, respectively. Negative valence increased significantly after induction in VRS group only. Arousal decreased significantly in VRS group only, after ER only. Dominance decreased & increased non-significantly after induction & ER in both groups, respectively. Physiological regulation: Θ activation in brain regions associated with emotion processing increased significantly only in VRS group. No association in other EEG frequencies. |  | **-** |
| 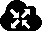 Ruiz-Ariza 2018 (72) | 1. Self-control scale (self) 2. Emotional intelligence scale (self) |  |  | ER: In AR outdoor quest, self-control decreased & emotional intelligence increased non-significantly. | *Social skills, executive function* | 5.4% |
| 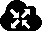 ^a^Yuan 2018 (83) | ER & expression test (parent) |  |  | ER: In immersive group VR emotion & social skill practice scenarios, ER & expression increased non-significantly. | Trainer guidance & debrief/*Social cognition, social skills* | **-** |
| ^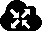 a^Ip 2018 (66) | ER & expression test (parent) |  |  | ER: In immersive individual VR emotion & social skill practice scenarios, ER & expression increased significantly, with a small effect (*g* = 0.38). | Observation, trainer guidance, debrief & worksheets/ *Social cognition, social skills* | 2.25% |
| ^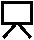 a^Carroll 2016 (54) |  | 1. Positive emotion scale (self) 2. Negative emotion scale (self) |  | Emotion experience: In multimedia modular programme, intensity of positive & negative emotions decreased significantly, with small effects (*g* = -0.47; *g* = -0.43), respectively. | Classwork, homework & finale celebration/ *Social cognition, social skills* | 3.4% |
| 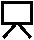 ^a^Houghton 2017 (65) |  | 1. Positive emotion scale (self) 2. Negative emotion scale (self) |  | Emotion experience: In multimedia modular programme, intensity of positive emotions decreased significantly, with a large effect (*g* = -1.39). Intensity of negative emotions decreased non-significantly. | Classwork, homework & finale celebration/  *Social cognition, social skills* | **-** |
| 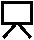 ^a^Carroll 2020 (55) | Socio-emotional competence scale (teacher) | Internalising scale (teacher) |  | ER: In multimedia modular programme, socio-emotional competence increased significantly, with a small effect (*g* = 0.17). Emotion experience: Internalising decreased non-significantly. | Classwork, homework & finale celebration/ *Social cognition, social skills* | **-** |

^a^=continuance of existing treatment unclear

^b^=continuance of existing treatment not permitted

^c^=continuance of existing treatment permitted
