# Supplementary material for: Digital Interventions for Emotion Regulation in Children and Early Adolescents: Systematic Review and Meta-analysis
Source: JMIR Serious Games. 2022 Aug 19;10(3):e31456. doi: 10.2196/31456 (PMC9440412; doi:10.2196/31456)
Supplement: Multimedia Appendix 11 [file games_v10i3e31456_app11.docx]

Online Supplementary Material Eleven.

Emotion regulation digital intervention feasibility matrix.

*Note.* This table includes the feasibility outcome summaries for all included studies, where feasibility data is available. In studies included in the meta-analytic component, between group feasibility data presented if available, with significance information.
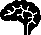
=biofeedback;
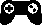
=digital game;
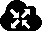
=virtual reality/augmented reality;
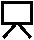
=programme/ multimedia. ER=emotion regulation. **-**=not reported. Red shading=high ROB. RPG=role player game; ADHD=attention deficit hyperactivity disorder; VR=virtual reality; AR=augmented reality. HR=heart rate; GSR= galvanic skin response. NF=neurofeedback; BF=biofeedback. Additional feasibility information provided below information borne out of feasibility measures. See online Supplementary Material 7 for details of feasibility measures.

| ID | Dropout % & reasons | Measure & ROB | Report | Outcome summary & additional information |
| --- | --- | --- | --- | --- |
| 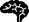 Cohen 2016 (56) | 9.5% |  |  |  |
| 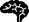 Torrado 2017 (79) | **-** | Field notes & comments | Researcher/caregiver | In HR threshold alert & personalised ER prompt providing smartwatch, one participant demonstrated 20/30 unsuccessful uses due to ignoring alert when in difficult situation & not knowing how to use watch on days 1-3. High level of support required on days 1-3 to use watch & ER. Activated alerts due to excessive excitement–caregiver noted helpful as excessive excitement caused emotional outbursts. Ongoing support required to associate prompts to music video ER strategy on computer. One participant who experienced light/sound sensitivity activated 54 ER prompts. 31/54 unsuccessful uses due to not knowing how to use watch on days 1-4 & unable to use due to high level of distress*.* High level of support required on days 1-4. Lowered hands from ears on days 6, 7 & 9 when noticed alert (hands on ears a lot due to loud noises from classmates).  Both participants able to interact successfully & autonomously with watch by end of intervention. Also acted as distractor when in difficult situation. |
| 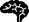 Lackner 2016 (68) | 9% |  |  |  |
| ^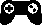 a^Rogel 2020 (71) | 22% ITT: Personal/family/ communication issue, group assignment displeasure |  |  |  |
| 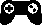 David 2019 (58) | 7%: Withdrew |  |  | In 2D ER game, maximum 50min completion time per mini-game determined in pre-study testing with *N*=5 youths. Missed levels played in next session in current study. |
| 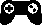 David 2020 (59) | 18.8%: Did not complete first intervention task |  |  | In 2D ER game, wireless Emotiv EPOC 14-channel EEG technical issues severely affected EEG signal quality. |
| 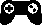 Rodriguez 2015 (70) | **-** |  |  | In VR 3D frustration induction & deep breathing/focused attention ER game, X3 engagements required to determine stability of ER ability. |
| ^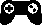 a^Antle 2018 (49) | 9%: Left participating school | Behavioral assessment scale & open questions | School staff | In a EEG-NF, body relaxation/deep-breathing ER game, learnt skills used in classroom & playground. |
| 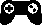 Lutz 2014 (69) | **-** | Verbal feedback  Observations | Clinician  Clinician | In 2D HRV BF deep breathing & positive focus ER game, easy integration in therapy sessions but some reports of BF set-up & explanation difficulties. No real-life practice of learnt ER strategies when ER strategy/game novelty declined. |
| 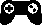 Schuurmans 2018 (75) | 34% ITT: Discharged from participating clinic, re-placed, behavioural improvement/ decline, refused treatment | Game tutorial compliance scale  Open-answer question on skill use | Self  Self | In 3D immersive HR BF fear, frustration & anger induction ER game, high compliance in anger: positive self-talk (*M* = 5.76/7) & guided imagery (*M* = 5.95), frustration: muscle relaxation (*M* = 6.12/7), fear: deep-breathing (*M* = 6.06/7) mini-game tutorials.  Deep-breathing & positive thinking strategies were used most in real-life. |
| 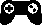 Scholten 2016 (73) | 8.7% ITT |  |  |  |
| 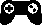 Schoneveld 2016 (46) | 25.7% ITT |  |  |  |
| 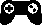 Schoneveld 2018 (47) | 12% ITT: Time issues in control group |  |  | 87% (*n* = 64) intervention group & 91% (*n* = 66) control group attended at least 5 or 7 sessions, respectively. |
| 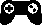 Schoneveld 2020 (74) | *See 21 ES2018* |  |  |  |
| 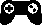 Wijnhoven 2020 (45) | 32% |  |  | 73% (*n* = 39/53) intervention group & 80% (*n* = 45/56) control group attended 6 sessions. |
| ^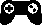 a^Beaumont 2008 (50) | **-** |  |  | In 3D ER, social cognition & social skill RPG with allied group-based learning sessions, pre-study testing conducted with 8 ASD/healthy youth to check engagement & difficultly. |
| ^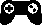 a^Beaumont 2015 (51) | 1.4%: Left participating school |  |  |  |
| ^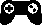 a^Sofronnoff 2017 (78) | 32%: Personal/family issue, time constraints, engagement/ motivation issues |  |  | In 3D ER, social cognition & social skill RPG with allied home-based learning sessions delivered by parents, dropout parents younger with lower education level & higher ASD traits than non-dropouts. 10-week program took parents 12-18 weeks to deliver. |
| ^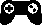 a^Shum 2019 (76) | 29.2% |  |  | In 2D modular ER, social cognition, social skill & mental health game with allied class-based learning, 68.9% (*n* = 182/264) participants reached intervention completion rate of >50%. |
| 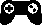 Carlier 2020 (53) | 40%: Illness, game too easy | Engagement game data  Interview | Parent | In 2D guided imagery & deep breathing ER & non-therapeutic game, platformer non-therapeutic mini-game was most played in 2/3 participants (44% & 92% gameplay), but 0% completion. Memory non-therapeutic mini-game was most played in 1/3 participants (41% gameplay) & second in 2/3 participants (23% & 4% gameplay). Guided imagery & breathing therapeutic mini-games were least played in all participants. Distracted by ‘owning’ smartphone device on which intervention played (2 youths given smartphone by parents–parents did not use parent tracking app). WIFI issues prevented synchronisation of parent tracking app data in one parent. Parents did not track non-spontaneous engagement. Engagement encouraged by parents only when participant calm.  High engagement in platformer mini-game in one participant attributed to potential provocation of repetitive behaviour. Anxiety measures completed incorrectly. |
| 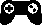 Amon 2008 (48) | **-** | Diary  Game experience scale | Parent  Parent | In HR & GSR BF breathing strategy ER game, dizziness, emotional outbursts, tiredness, low appetite & hyperactivity experienced by 8-25% (*n* = 2-6/24) youths diagnosed with ADHD did not change significantly over intervention. No symptoms in healthy youth.  54%, (*n* = 13/24) diagnosed youths practiced breathing technique in real-life by end of intervention. |
| 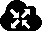 Wrzesien 2015 (82) | **-** | VR avatar identification scale  VR presence scale | Self  Self | In immersive VR frustration induction with avatar that modelled emotions, behaviours & ER, youth did not identify physically/behaviourally with self-representing (*M* = 3.47/8; 3.17/8) or neutral (*M* = 2.02/8; 2.39/8) avatar. Did not identify emotionally with self-representing (*M* = 3.39/8) but did somewhat with neutral (*M* = 3.42/8) avatar.  No/neutral presence in self-representing (*M* = 5.83/9) & neutral (*M* = 5.09/9) VR. |
| ^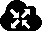 a^Ruiz-Ariza 2018 (72) | 5.4%: Stopped using application |  |  |  |
| ^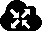 a^Yuan 2018 (83) | **-** | Communication log | Parents & teachers | In immersive group VR emotion & social skill practice scenarios, trainer facilitated understanding of audio/visual aids. Trainer provided behavioural/emotional support that permitted use of VR goggles successfully & without distress after 3/12 sessions. Briefing & debriefing sessions essential to generalise learnt skills to real-life. |
| ^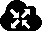 a^Ip 2018 (66) | 2.25%: Expectations not met, time issues |  |  |  |
| ^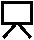 a^Carroll 2016 (54) | 3.4%: Left participating school, withdrew | Verbal feedback | Facilitator | In multimedia modular programme, literacy requirement/content complexity too high for <8 years. Time constraints negatively affected delivery. Recommendations: Teachers to have formal intervention training. Make contingency plans for absentees. Reduce text in manual. Stories promoted engagement & should involve whole-class. |
| 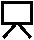 ^a^Houghton 2017 (65) | **-** | Verbal feedback | Facilitator | In multimedia modular programme, literacy requirement/content complexity too high. Time constraints negatively affected delivery. Manual detailed & easy to follow. |
| ^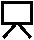 a^Smith 2018 (77) | 0 reported |  |  | In online programme, due to minimal time available to fill in scales, authors created short scales. |

^a^=intervention also trains other skills.
