# Supplementary material for: Digital Interventions for Emotion Regulation in Children and Early Adolescents: Systematic Review and Meta-analysis
Source: JMIR Serious Games. 2022 Aug 19;10(3):e31456. doi: 10.2196/31456 (PMC9440412; doi:10.2196/31456)
Supplement: Multimedia Appendix 12 [file games_v10i3e31456_app12.docx]

Online Supplementary Material Twelve.

Emotion regulation digital intervention acceptability matrix.

*Note.* This table includes the acceptability outcome summaries for all included studies, where acceptability data is available. In studies included in the meta-analytic component, between group acceptability data presented if available, with significance information.
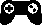
=digital game;
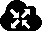
=virtual reality/augmented reality;
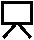
=programme/multimedia. ER=emotion regulation. Green shading=low ROB; red shading=high ROB. ADHD=attention deficit hyperactivity disorder; CBT=cognitive behavioural therapy; VR=virtual reality; AR=augmented reality. HR=heart rate; GSR= galvanic skin response. NF=neurofeedback; BF=biofeedback. See online Supplementary Material 7 for details of acceptability measures.

| ID | Measure & ROB | Report | Outcome summary |
| --- | --- | --- | --- |
| 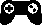 Vara 2016 (80) | Game likeability & usefulness scale | Self | In VR 3D frustration induction & deep breathing ER game using different devices, higher induction likeability in smartphone (*M* = 4.19/5) & camera (*M* = 4.3/5) device than computer (*M* = 2.88/5). Device type did not affect breathing mini-game likeability (*M* = 3.6-3.9/5). Higher usefulness of breathing strategy in smartphone device (*M* = 3.85/5) than camera (*M* = 3.7/5) & computer (*M* = 3.12/5). |
| ^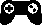 a^Antle 2018 (49) | Focus group, observations, written reports & email updates | School staff | In an EEG-NF, body relaxation/deep-breathing ER game, no usability issues. All participants easily learned to use their bodies to implement strategies & successfully play all mini-games. Real-time calibration (making game easier by lowering relaxation threshold/decreasing hold time) required in 1/2 ER mini-games. |
| 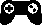 Kahn 2013 (67) | Game helpfulness scale | Self | In a 2D HR BF deep breathing ER game, high helpfulness (*Median* = 5-6/7). |
| 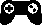 Lutz 2014 (69) | Verbal feedback Verbal feedback  Observations | Self  Clinician  Clinician | In 2D HRV BF deep breathing & positive focus ER game, repeat game sessions requested. Clinicians inspired to expand on strategies dependent on needs, preferences & treatment style. Created vibrant learning community. Some performance anxiety, especially when losing previously won rewards. |
| 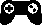 Schuurmans 2018 (75) | Game appeal, usefulness & likeability scale | Self | In 3D immersive HR BF fear, frustration & anger induction ER game (*intervention group only)*, *h*igh appeal to oneself (*M* = 4.53/5) & other children (*M* = 4/5). Liked that a digital game is an intervention (*M* = 3.88/5) & very useful in daily life (*M* = 4.53/5). |
| **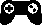** Schoneveld 2016 (46) | Game difficulty, flow, appeal & relevance scale | Self | In 3D immersive EEG NF anxiety induction ER game, moderate difficulty (*M* = 2/4), flow (*M* = 1.94/4), appeal (*M* = 1.9/4) & appeal to other children (*M* = 2.29/4). Low relevance (*M* = 1.68/4). Non-significant difference in difficulty, relevance & appeal to other children. Significant difference in appeal & flow. *Higher rating in commercial control game* *relative to intervention.* |
| **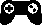** Schoneveld 2018 (47) | Game difficulty, fun & relevance scale | Self | In 3D immersive EEG NF anxiety induction ER game, moderately fun (*M* = 2.35/5) & fun for other children (*M* = 2.61/5). Low difficulty (*M* = 1.85/5) & relevance (*M* = 2.13/5). Non-significant difference in difficulty, fun & fun to other children. Significant difference in relevance. *Higher rating in control group CBT relative to intervention.* |
| 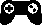 Carlier 2020 (53) | Game emotion scale  Interview | Self  Parent | In 2D guided imagery & deep breathing ER & non-therapeutic game, mainly happy in non-therapeutic mini-games. One participant very angry in platformer non-therapeutic mini-game. Breathing mini game boring & difficult. One participant refused to play platformer non-therapeutic mini game as too difficult. One parent reported platformer non-therapeutic mini-game too difficult in lowest difficulty level & guided imagery mini-game became too easy. Manually switching between difficulty levels reported as a nuisance. |
| 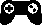 Amon 2008 (48) | Game experience scale | Parent | In HR & GSR BF breathing strategy ER game, most ADHD (70.8%, *n* = 17/24) & healthy (83.3%, *n* = 10/12) youths experienced moderate difficultly & just over half of ADHD youths (58.3%, *n* = 14/24) still found game difficult by end of intervention. Most healthy youths experienced low difficulty by end of intervention (58.3%, *n* = 7/12). *Benefit of using techniques in real-life unclear.* |
| 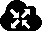 Wrzesien 2015 (82) | VR education & fun scale | Self | In immersive VR frustration induction with avatar that modelled emotions, behaviours & ER, youths reported moderate fun & educational impact. Self-representing & neutral avatar likeable. |
| ^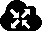 a^Ruiz-Ariza 2018 (72) | AR happiness, danger, helpfulness, motivation, satisfaction & further play dichotomous questions | Self | In AR outdoor quest, youths felt happy (54.5%, *n* = 48/87), motivated to go out (56.8%, *n* = 50/87), intervention is dangerous (72.7%, *n* = 64/87) helped to make friends (52.3%, *n* = 46/87). Willing to keep playing (63.6%, *n* = 56/87) & test new versions (77.3%). Males played to have fun, females played due to boredom. Males reported greater satisfaction than females. |
| ^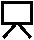 a^Carroll 2016 (54) | Education & likeability scale Verbal feedback | Self  Facilitator | In multimedia modular programme, youths liked the programme ‘A lot’ or ‘Very, very much’. Learned ‘A lot’ or ‘Very, very much’.  Stories promoted enjoyment - they should involve the whole-class. Content relevant & had positive impact. |
| ^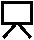 a^Houghton 2017 (65) | Education & likeability scale Verbal feedback  Programme quality scale | Self  Facilitator  Facilitator | In multimedia modular programme, youths liked the programme ‘Very much’ or ‘Quite a lot’. Learned ‘Very much’, ‘Quite a lot’ or ‘Some things’.  Programme flowed well. Materials acceptable to youths; used each week & had positive impact.  High quality programme. |

^a^=intervention also trains other skills
